# Predicting cardiovascular disease in peritoneal dialysis patients using glycated proteomics and machine learning: A prospective multicentre study

**DOI:** 10.1002/ctm2.70889

**Published:** 2026-10-27

**Authors:** Ji Ji, Zhaoying Nie, Na Jiang, Ting Xie, Lei Zhang, Xiaoyan Liu, Min Lu, Bo Xiang, Weiwei Wu, Xiaotian Jiang, Lin Zhang, Jun Ji, Jianzhou Zou, Xiaoqiang Ding, Haojie Lu, Xiaofang Yu

**Affiliations:** ^1^ Department of Nephrology Zhongshan Hospital, Fudan University Shanghai China; ^2^ Shanghai Medical Center of Kidney Shanghai China; ^3^ Shanghai Key Laboratory of Kidney and Blood Purification Shanghai China; ^4^ Shanghai Institute of Kidney and Dialysis Shanghai China; ^5^ Department of Nephrology Renji Hospital, School of Medicine, Shanghai Jiaotong University Shanghai China; ^6^ Division of Nephrology Minhang Hospital, Fudan University Shanghai China; ^7^ Department of Nephrology Shanghai Baoshan District Wusong Central Hospital (Wusong Branch, Zhongshan Hospital Affiliated to Fudan University) Shanghai China; ^8^ Department of Nephrology Second Hospital, Dalian Medical University Dalian China; ^9^ Department of Chemistry Liver Cancer Institute, Zhongshan Hospital, Fudan University Shanghai China; ^10^ Institutes of Biomedical Sciences and NHC Key Laboratory of Glycoconjugates Research, Fudan University Shanghai China

**Keywords:** cardiovascular disease, glycated proteomics, machine learning, peritoneal dialysis

## Abstract

**Background:**

Cardiovascular disease (CVD) is the leading cause of death among peritoneal dialysis (PD) patients, yet PD‐specific biomarkers and prediction models remain limited. This study aimed to identify glycated peptide biomarkers associated with CVD in PD patients and develop a prediction model integrating glycated proteomics and clinical indicators using machine learning.

**Methods:**

In Phase I, a matched discovery cohort (48 PD patients with CVD and 48 without CVD) was used to identify glycated peptides by liquid chromatography‐tandem mass spectrometry (LC‐MS/MS), with glmnet used to prioritise candidate biomarkers. In Phase II, candidate biomarkers were quantified in a prospective internal derivation cohort (*n* = 240) and a multicentre external validation cohort (*n* = 102). The combined model was developed using *F*‐test feature selection and elastic‐net logistic regression. Its predictive performance was compared with three published cardiovascular risk models. Patients were stratified using a training‐derived Youden cutoff.

**Results:**

In the discovery cohort, 257 glycated peptides were differentially abundant between groups. Four candidate biomarkers were prioritised, and two peptides, GEAFTLK(g)ATVLNYLPK and K(g)QHLFVK, were retained in the final model together with six clinical indicators: phosphate, diabetes, total Kt/V, albumin, cardiac troponin T, and glycated albumin. The combined model achieved an area under the receiver operating characteristic curve (ROC‐AUC) of 0.784 in the training set, 0.781 in the internal testing set, and 0.822 in the external validation cohort, respectively. Using the training‐derived cutoff of 0.373, higher 1‐year CVD incidence was observed in the high‐risk group in both validation cohorts. The combined model also achieved higher ROC‐AUCs than the published cardiovascular risk models.

**Conclusion:**

We developed and externally validated a PD‐specific CVD prediction model by integrating glycated peptide biomarkers with clinical indicators, which may facilitate earlier risk stratification and targeted prevention.

## Graphical abstract

Graphical Headlights

1. Glycated proteomics identified candidate cardiovascular biomarkers in peritoneal dialysis patients.

2. A machine learning model integrated two glycated peptides with six clinical indicators.

3. The model achieved ROC‐AUCs of 0.781 in the internal testing set and 0.822 in multicentre external validation.

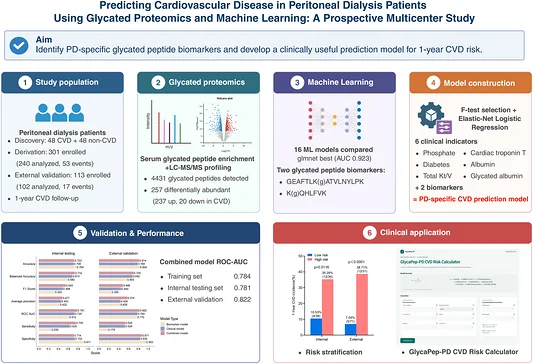

## 1 INTRODUCTION

The worldwide burden of chronic kidney disease (CKD) includes millions of deaths annually, and CKD is projected to rank fifth among causes of death by 2040.^1^, ^2^ For this progressive, irreversible disease, effective curative treatment remains unavailable. Progression to end‐stage renal disease leaves renal replacement therapy as the only treatment option, with either peritoneal dialysis (PD) or haemodialysis (HD) currently constituting its main modality.

The survival benefits of HD and PD are accompanied by a continuing burden of severe complications in dialysis patients, with cardiovascular disease (CVD) being the most common complication and the chief cause of mortality. Among deaths recorded in the United States in 2023, the proportions attributed to CVD were 58.6% for HD patients and 54.6% for PD patients.^3^ Compared with HD, uremic solutes and fluid are removed more steadily with PD, a difference that could theoretically reduce the risks of cardiovascular events and mortality.^4^ However, PD may increase cardiovascular risk due to inadequate volume removal, persistent chronic inflammation of the peritoneum and fluctuations in blood glucose levels.^5^, ^6^, ^7^ Early prediction and diagnosis of CVD, followed by timely intervention, may help reduce cardiovascular mortality. A variety of biomarkers have been identified for predicting CVD, such as N‐terminal pro‐B‐type natriuretic peptide (NT‐proBNP), soluble suppression of tumourigenicity 2, and angiopoietin‐2, some of which have been applied in clinical practice. However, these biomarkers still have limitations, including insufficient sensitivity and specificity, and their levels are easily influenced by residual renal function and dialysis status.^8^, ^9^, ^10^, ^11^

Protein glycation is a passive, pathological modification that commonly occurs during metabolic disturbances such as hyperglycaemia and aging. It refers to a series of non‐enzymatic reactions in which reducing sugars covalently react with amino groups on proteins, ultimately leading to the irreversible formation of advanced glycation end products (AGEs). This process involves two stages: an initial stage in which early glycation products (EGPs) are formed reversibly, followed by a later stage in which AGEs are generated irreversibly.^12^, ^13^ AGEs are elevated in CKD and have been linked to cardiovascular risk and mortality even in the absence of hyperglycaemia, consistent with their pro‐inflammatory and pro‐oxidative properties.^14^ In the PD population, a higher AGE burden was independently associated with cardiovascular morbidity as well as carotid atherosclerosis and vascular calcification.^15^, ^16^

However, AGEs are late‐stage and irreversible products that mainly reflect long‐term metabolic stress, limiting their utility for early risk assessment. Thus, early glycation products may be more suitable for early CVD prediction. Current evidence has suggested that albumin‐derived EGPs can serve as biomarkers of diabetic vascular complications.^17^ In our previous study, we identified 17 differential glycated peptides in HD patients and validated four of them for CVD.^18^ However, this study identified biomarkers through traditional bioinformatics analysis and literature review, which was a key limitation. Moreover, in PD patients, high glucose exposure from peritoneal dialysate can exacerbate protein glycation in vivo, potentially contributing to the development and progression of CVD.^19^, ^20^ Therefore, we aimed to identify glycated peptide biomarkers for CVD prediction in PD patients using machine learning(ML) strategies, integrate them with traditional CVD risk factors to construct a risk prediction model, and validate the model across multiple PD centres.

## 2 METHODS

### 2.1 Study design and population

The study linked biomarker discovery with prospective model development and validation in two phases (Figure 1). The first phase was a matched case‐control analysis within an established cohort of PD patients at Zhongshan Hospital, Fudan University, using stored serum samples collected before CVD happened and used to define cases. Cases and controls were selected retrospectively according to subsequent CVD outcomes, forming the discovery cohort for screening candidate CVD biomarkers. The second phase was a prospective study comprising two independent cohorts, including a single‐centre internal derivation cohort and a multicentre external validation cohort, which were used for the development and validation of the CVD prediction model.

**FIGURE 1:**
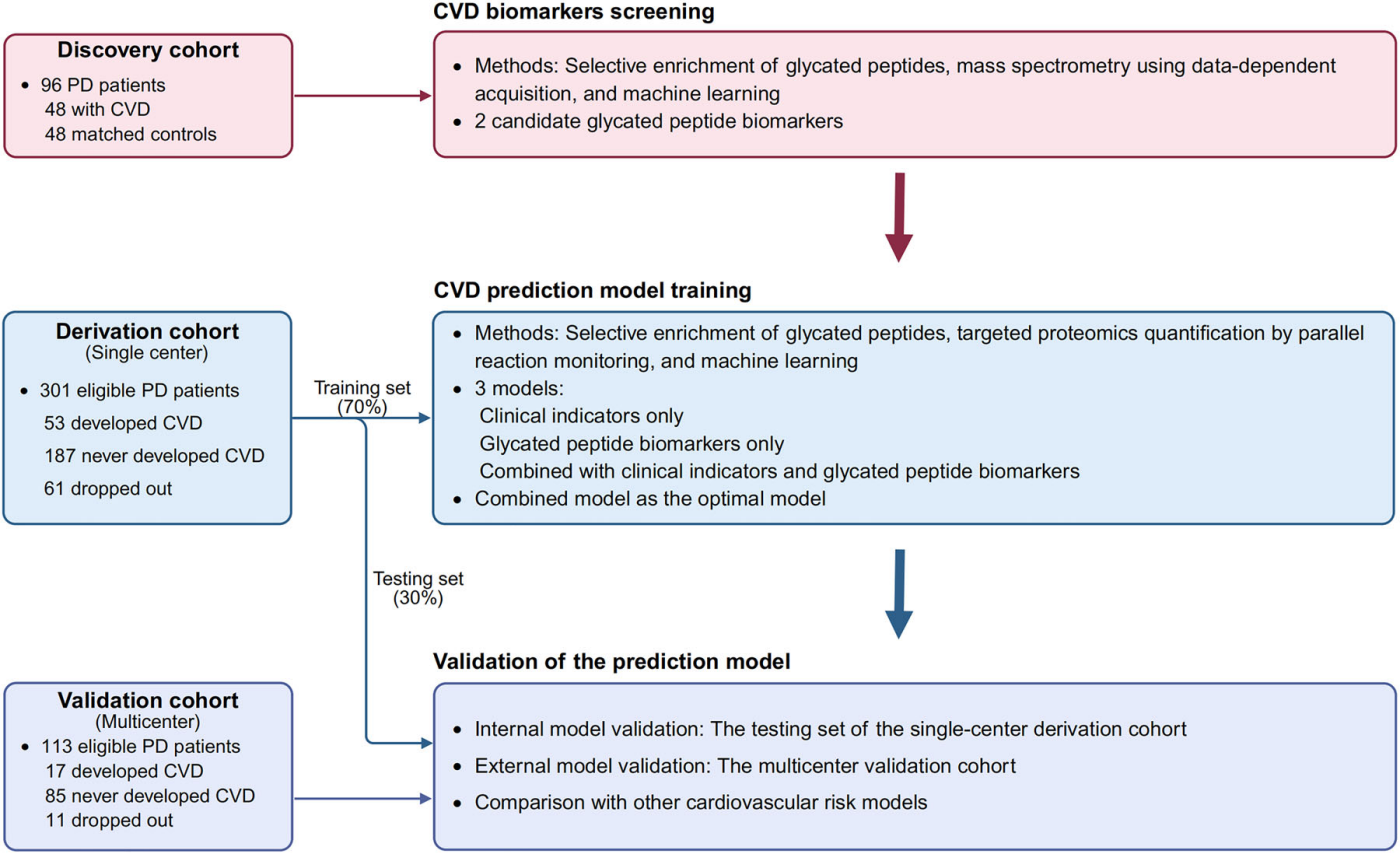
Study design. PD, peritoneal dialysis; CVD, cardiovascular disease.

For the discovery cohort, patients who received regular PD treatment at Zhongshan Hospital, Fudan University, between June 2019 and December 2021 and met the criteria for CVD were enrolled. CVD was defined as the presence of acute myocardial infarction, coronary artery disease, cerebrovascular accident, transient ischaemic attack, sudden cardiac death, heart failure, and peripheral artery disease. The inclusion criteria were as follows: (1) age between 18 and 80 years; (2) receiving regular PD for more than 6 months. The exclusion criteria were as follows: (1) kidney transplantation prior to dialysis; (2) combined therapy with PD and HD; (3) concomitant malignant tumours or autoimmune diseases; (4) chronic infection or an episode of infection within 3 months prior to enrolment; (5) new or distinct recurrent cardiovascular diseases occurred within the 6 months before sample collection. Ultimately, 48 eligible patients were included in the case group, and one control was matched to each case in a 1:1 ratio based on age, sex, and dialysis vintage.

To establish the derivation cohort, we prospectively enrolled patients who received regular PD therapy at Zhongshan Hospital, Fudan University, between January 2022 and September 2024. For the multicentre external validation cohort, patients undergoing regular PD between May 2024 and October 2024 were enrolled from Renji Hospital, School of Medicine, Shanghai Jiaotong University; Minhang Hospital, Fudan University; Wusong Central Hospital of Baoshan District; and Second Hospital, Dalian Medical University. Both cohorts used the same inclusion and exclusion criteria as the discovery cohort. A total of 301 eligible patients were included in the derivation cohort, and 113 were included in the external validation cohort. Follow‐up lasted 1 year for all participants. During follow‐up, patients who were lost to follow‐up, died from non‐cardiovascular causes, converted to HD or combined PD and HD therapy, or underwent kidney transplantation were considered dropouts. The primary outcome was the incidence of newly diagnosed or distinct recurrent CVD, defined consistently with the criteria used for the discovery cohort. Participants classified as dropouts were excluded from the analyses. Clinical data were complete among analysed participants, and no imputation was performed.

Approval was granted by the Ethical Committee of Zhongshan Hospital, Fudan University, under approval numbers B2017‐108R and B2022‐161R. Written informed consent was obtained from every participant.

### 2.2 Sample and data collection

The sample size was estimated based on the preliminary predictive performance of the four candidate glycated peptide biomarkers, which achieved a combined area under the receiver operating characteristic curve (ROC‐AUC) of 0.809 in the discovery cohort. Assuming an anticipated ROC‐AUC of 0.75, a two‐sided α of 0.05, 90% power, and an expected 1‐year CVD incidence of approximately 20% in PD patients, at least 200 evaluable participants were required for model development. Considering a potential dropout rate of 20%, the planned enrolment of approximately 250 patients was considered.

At enrolment, fasting blood was drawn before PD exchange. Serum was obtained by centrifugation at 3000 g for 10 min and immediately stored at –80°C pending analysis. Baseline characterisation covered sex, age, dialysis vintage, body mass index, hypertension, diabetes, CVD history, smoking status, medication use during the previous 3 months, and blood pressure. The laboratory assessment recorded red blood cells, haemoglobin, platelets, white blood cells, neutrophils, total bilirubin, direct bilirubin, total protein, albumin, fasting blood glucose, glycated haemoglobin (HbA1c), glycated albumin (GA), alanine aminotransferase, aspartate aminotransferase, lactate dehydrogenase, serum creatinine, blood urea, uric acid, estimated glomerular filtration rate, triglycerides, total cholesterol (TC), low‐density lipoprotein cholesterol (LDL‐C), high‐density lipoprotein cholesterol (HDL‐C), calcium, phosphorus, serum iron, transferrin saturation, ferritin, C‐reactive protein, intact parathyroid hormone (iPTH), NT‐proBNP, cardiac troponin T (cTnT), and D‐dimer. Urine volume and the peritoneal, renal, and total components of Kt/V and creatinine clearance rate (Ccr) characterised dialysis‐related status. Imaging measures comprised carotid artery intima‐media thickness (IMT), left ventricular ejection fraction, and left ventricular end‐diastolic diameter. These clinical measurements also constituted the candidate indicators for prediction‐model construction.

### 2.3 Sample preparation for mass spectrometry analysis

Sample preparation began with reduction of 500 µg of serum protein in 75 mM sodium cyanoborohydride (Sigma‐Aldrich, USA) for 4 h at 37°C. Desalting was then carried out in 10 kDa ultrafiltration tubes (Merck Millipore, Germany) with 25 mM ammonium bicarbonate (Sigma‐Aldrich, USA). A subsequent reduction step used 10 mM dithiothreitol (Sigma‐Aldrich, USA) for 1 h at 56°C. This was followed by alkylation with 20 mM iodoacetamide (Sigma‐Aldrich, USA) for 45 min at room temperature, protected from light. Ultrafiltration removed the remaining reagents. To release N‐glycans, the proteins were incubated overnight at 37°C with 1 unit of PNGase F (New England Biolabs, USA). Following a further ultrafiltration step to remove the released glycans and low‐molecular‐weight impurities, the protein solution was recovered.

The recovered protein solution was denatured by heating for 10 min at 100°C, then diluted to 1 µg/µL. Digestion proceeded overnight at 37°C after addition of trypsin (Beijing Shengxia Proteins Scientific Ltd., China) at a trypsin‐to‐protein mass ratio of 1:50. Glycated peptides were subsequently enriched using Affi‐Gel boronate media (Bio‐Rad Laboratories, USA) during 16 h of shaking at 1100 rpm and 37°C. Sep‐Pak C18 columns (Waters, USA) were used to desalt the enriched peptides. The resulting eluates were lyophilised and kept at –80°C. Before analysis, peptide quantification was performed, and the lyophilised samples were dissolved to 0.5 µg/µL.

### 2.4 Mass spectrometry by data‐dependent acquisition

For nanoscale liquid chromatography‐tandem mass spectrometry (LC‐MS/MS), the analytical platform combined an EASY‐nLC 1200 with an Orbitrap Exploris 480 (Thermo Fisher Scientific Inc., San Jose, CA). Chromatographic separation used a 40‐cm column supplied by Kyoto Monotech (Kyoto, Japan), with 5 µL of peptide solution injected for each 60‐min acquisition. The acquisition range was *m*/*z* 350–1600, and the resolution setting was 60k at *m*/*z* 200. Precursor fragmentation used higher‐energy collisional dissociation at 29% normalised collision energy. MS/MS acquisition employed a resolution of 60 000 at *m*/*z* 200, an automatic gain control target of 75%, and a maximum injection time of 120 ms. PEAKS Online (Bioinformatics Solutions Inc.) was used to search all data files against the Homo sapiens database downloaded from UniProt in April 2023.

### 2.5 Targeted peptide quantification by parallel reaction monitoring

To validate the results of data‐dependent‐acquisition‐based proteomic analysis, we identified four candidate glycated peptide biomarkers, which were then quantified using a parallel reaction monitoring (PRM)‐based LC‐MS/MS method. For PRM experiments, four heavy isotope‐labelled standard glycated peptides with N‐terminal dimethylation and lysine glycation (synthesised by China Peptides Co., Ltd.) were spiked into the protein samples prior to digestion. All other experimental procedures were performed as described above.

Selection of precursor/product ion transitions, optimisation of collision energies, and PRM data acquisition were performed on Orbitrap Exploris 480. The parameters were the same as described above, and targeted peptide quantification by PRM was performed according to the peptide list (Table S1). All data files were imported into Skyline Daily (version 25.1.0). Using the heavy isotope–labelled standard glycated peptides as internal standards, the concentrations of the target glycated peptides were determined based on the peak area ratios to the corresponding standards. Peptide concentrations were expressed in pg/µL

### 2.6 Model construction and evaluation based on ML

In the first phase, to screen candidate CVD biomarkers from the glycated proteomics in the discovery cohort, we performed ML analyses using the mlr3verse package in R (version 4.3.0). The benchmark comprised 16 ML methods: generalised linear model network (Glmnet), neural network, generalised linear model boosting, multivariate adaptive regression splines, support vector machine (SVM), gradient boosting machine, extreme gradient boosting (XGBoost), generalised additive model boosting, linear discriminant analysis, light gradient boosting machine, random forest (RF), Gaussian process regression, naive Bayes, feedforward neural network, logistic regression (LR), and k‐nearest neighbours. Model performance in discriminating between the CVD and non‐CVD groups was assessed primarily using ROC‐AUC, accuracy, and *F*1 score. After a comprehensive comparison, the model with the best discriminatory performance was selected for biomarker screening. A random 7:3 allocation divided the discovery samples into training and testing sets, and the optimal model was trained on the training set and further evaluated on the testing set. Based on fourfold cross‐validation, the model coefficients of each feature variable, fold changes between CVD and non‐CVD groups, and statistical significance were considered for candidate biomarker selection.

In the second phase, to identify clinical predictive features and develop a CVD prediction model, patients in the derivation cohort were randomly split into a training set and a testing set at a 7:3 ratio. Model selection (comparison of algorithms and hyperparameter tuning) and feature selection were performed on the training set, and model performance was subsequently evaluated in the testing set. To systematically benchmark candidate modelling strategies, we evaluated a total of 34 model configurations using 56 clinical indicators in the training set under a nested cross‐validation framework. The inner layer used a threefold cross‐validation for hyperparameter tuning. The outer layer used a fivefold cross‐validation for unbiased model evaluation. The feature selection method was regarded as a hyperparameter in the tuning process. To leverage feature interactions, the glycated peptides were included in the feature selection process. Feature selection methods included *F*‐test, mutual information, L1 regularisation‐based selection, tree‐based feature importance, and recursive feature elimination. The classifiers included LR, elastic‐net logistic regression (ENetLR), SVM with a radial basis function kernel, RF, histogram‐based gradient boosting, and XGBoost. The number of selected clinical predictors was prespecified as six and enforced within each cross‐validation training fold. Model performance was assessed using ROC‐AUC, *F*1 score, balanced accuracy, area under the precision‐recall curve (PR‐AUC), precision, and recall. The best‐performing model was selected as the final prediction model which incorporated the six selected clinical indicators and two glycated peptide biomarkers identified in Phase I.

To evaluate model performance, we compared three models in the testing set: (1) a biomarker model using only glycated peptide biomarkers; (2) a clinical model using only the selected clinical indicators; and (3) a combined model integrating clinical indicators and biomarkers. Model performance was assessed using multiple complementary metrics, including specificity, sensitivity, ROC‐AUC, PR‐AUC, *F*1‐ score, balanced accuracy and accuracy.

### 2.7 Comparison with published cardiovascular risk models

The combined model was compared with three published cardiovascular risk models. The FRS was calculated using the 2008 lipid‐based model, which provides sex‐specific estimates of 10‐year general CVD risk based on age, systolic blood pressure (SBP), antihypertensive treatment, TC, HDL‐C, smoking status, and diabetes.^21^ The World Health Organization (WHO) CVD risk model was assessed using the 2019 laboratory‐based version for East Asia. This model incorporates age, sex, SBP, TC, smoking status, and diabetes.^22^ The PD‐specific nomogram developed by Xia et al. in 2017 incorporates age, history of CVD, diabetes, serum albumin, haemoglobin, C‐reactive protein, and 24‐h urine output.^23^

### 2.8 Statistical analysis

R (version 4.3.0) was used for Spearman's rank correlation analysis, ROC analysis, DeLong's test, selection of the Youden cutoff, decision curve analysis, calibration curve analysis, and Brier score analysis. Descriptive summaries reflected the type and distribution of each variable: normally distributed continuous data were summarised by the mean and standard deviation, non‐normally distributed continuous data by the median and minimum‐to‐maximum range, and categorical data by frequencies and percentages. Group comparisons used the Mann–Whitney *U* test for continuous measures and Fisher's exact test for categorical measures. Multiplicity adjustment employed Holm's step‐down procedure, and statistical significance was defined by a two‐sided *p* value below .05.

## 3 RESULTS

### 3.1 Candidate biomarker screening in discovery cohort

Figure 2A illustrates the study design of the first phase. The discovery cohort included 48 pairs of matched PD patients, comprising a CVD group and a non‐CVD group, and was used to screen glycated peptide biomarkers associated with CVD in PD patients (Table S2). A total of 4431 glycated peptides were identified, among which 1032 glycated peptides were commonly identified in both groups. Compared with the non‐CVD group, 237 glycated peptides were significantly upregulated, and 20 were significantly downregulated (Figure 2B).

**FIGURE 2:**
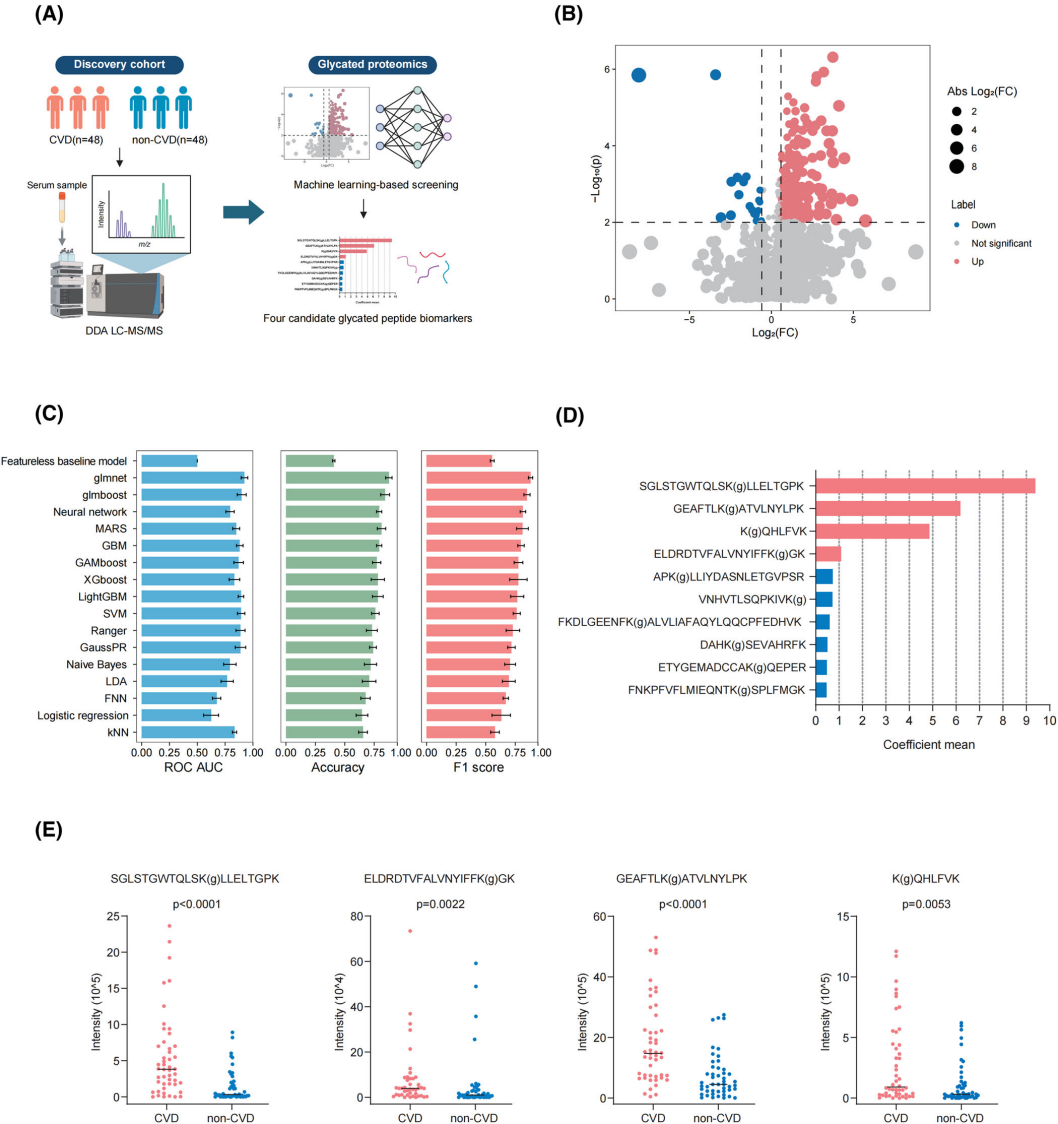
Glycated peptide biomarker screening in the discovery cohort. (A) The workflow of screening glycated peptide biomarkers in the discovery cohort. DDA, data‐dependent acquisition. (B) Volcano plot comparing the differential expression of glycated peptides between the CVD and non‐CVD groups. Glycated peptides with a fold change > 1.5 or < 0.67 and an adjusted *p* value < .01 were considered significantly differentially expressed. (C) Performance comparison of the 16 machine learning models used for biomarker screening. Glmnet, generalised linear model network; glmboost, generalised linear model boosting; MARS, multivariate adaptive regression splines; GBM, gradient boosting machine; GAMboost, generalised additive model boosting; XGBoost, extreme gradient boosting; LightGBM, light gradient boosting machine; SVM, support vector machine; GaussPR, Gaussian process regression; LDA, linear discriminant analysis; FNN, feedforward neural network; kNN, k‐nearest neighbours. (D) Feature‐importance ranking of glycated peptides selected by the glmnet model. (E) Relative intensity distribution of the four glycated peptides in the CVD and non‐CVD groups.

To identify candidate biomarkers, we compared 16 ML models (Figure 2C; Table S3). The glmnet model showed the best overall performance, with an ROC‐AUC of 0.923 (95% CI: 0.841–0.979), an accuracy of 0.875 (95% CI: 0.799–0.949), and an *F*1 score of 0.879 (95% CI: 0.828–0.929). The glmnet model ultimately selected 55 glycated peptides that contributed substantially in discriminating patients with CVD from those without CVD (Table S4). Based on the feature importance rankings, the top four peptides were chosen as candidate biomarkers, and they were SGLSTGWTQLSK(g)LLELTGPK from alpha‐1β‐glycoprotein, GEAFTLK(g)ATVLNYLPK from alpha‐2‐macroglobulin (α2M), K(g)QHLFVK from apolipoprotein B‐100 (ApoB) and ELDRDTVFALVNYIFFK(g)GK from alpha‐1‐antitrypsin (Figure 2D). Then, we established a targeted quantification method for glycated peptides. Since SGLSTGWTQLSK(g)LLELTGPK and ELDRDTVFALVNYIFFK(g)GK were below the limit of detection in many samples (Figure 2E; Table S5), GEAFTLK(g)ATVLNYLPK and K(g)QHLFVK were ultimately selected as the candidate biomarkers.

### 3.2 Targeted quantification of candidate biomarkers in the derivation cohort

Figure 3A illustrates the study design of the second phase. To validate the predictive performance of the candidate biomarkers and to develop a CVD prediction model, we prospectively enrolled 301 eligible PD patients from a single centre and followed them for 1 year. Ultimately, 240 patients were included, 53 of whom developed CVD. Table S6 presents the baseline characteristics of the patients in the derivation cohort. We performed absolute targeted quantification of the two candidate biomarkers and found that, compared with patients who did not develop CVD, those who developed CVD exhibited significantly higher levels of the two biomarkers (Figure 3B).

**FIGURE 3:**
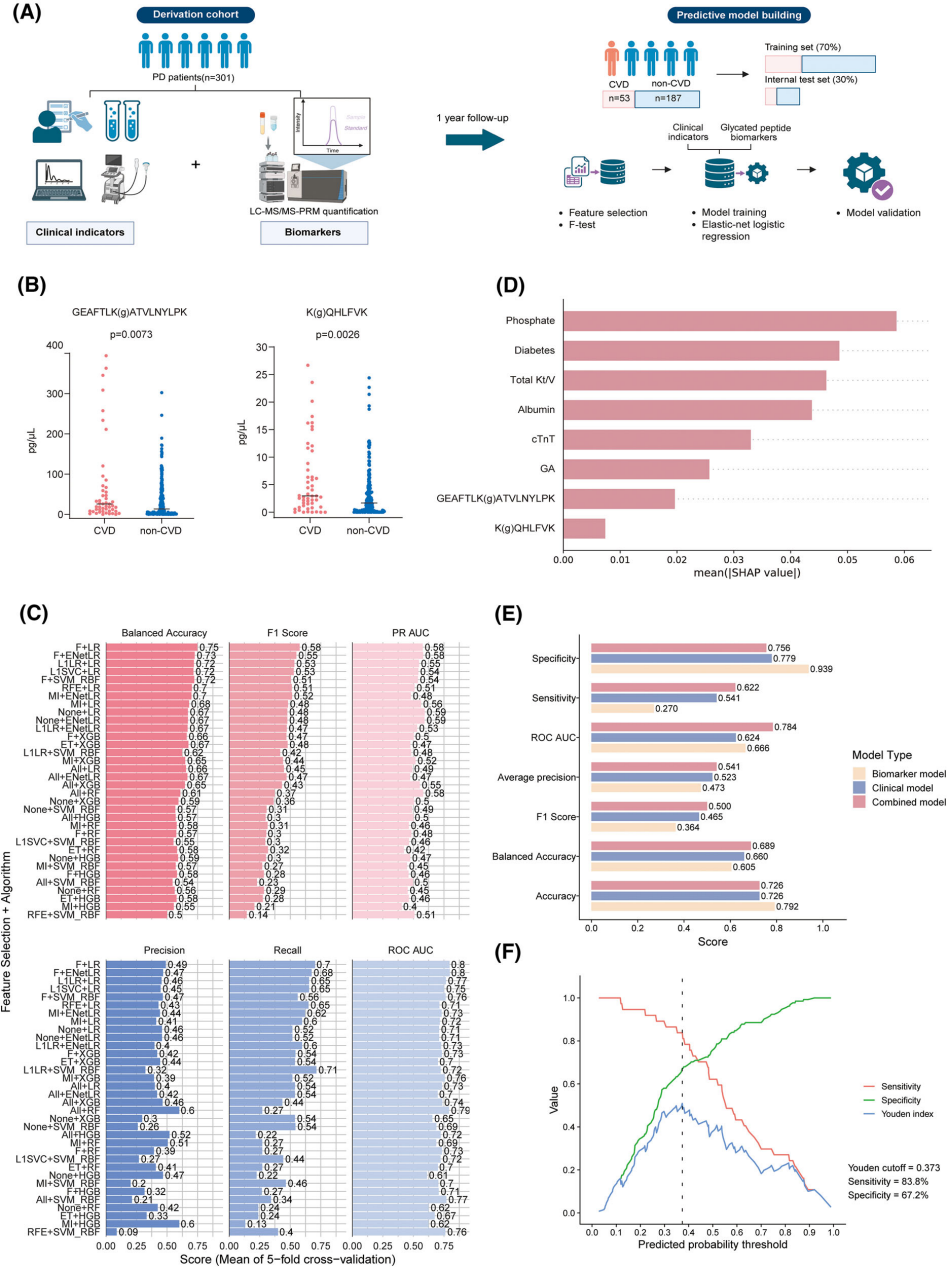
Model construction and evaluation in the derivation cohort. (A) The workflow of model construction and evaluation in the derivation cohort. PD, peritoneal dialysis; LC‐MS/MS, liquid chromatography–tandem mass spectrometry; PRM, parallel reaction monitoring. (B) Targeted quantification of the two biomarkers in the derivation cohort. (C) Performance comparison across 34 combinations of feature selection strategies and machine learning classifiers. F, *F*‐test; LR, logistic regression; ENetLR, elastic‐net logistic regression; L1LR, L1 regularisation; L1SVC, L1‐regularised support vector classifier; SVM‐RBF, support vector machine with a radial basis function kernel; RFE, recursive feature elimination; MI, mutual information; XGB, extreme gradient boosting; ET, tree‐based importance; RF, random forest; HGB, histogram‐based gradient boosting. (D) Bar plot ranking features by mean absolute SHAP values. SHAP, Shapley additive explanations. (E) Comparison of model performance among the biomarker model, clinical model, and combined model in the single‐centre training set. (F) Determination of the optimal risk stratification cutoff based on the Youden index.

### 3.3 Construction of combined model incorporating biomarkers and clinical indicators

To develop a model for predicting 1‐year CVD risk in PD patients, we integrated 56 clinical indicators with the two biomarkers and conducted a comprehensive benchmark analysis across 34 different model configurations to screen for features with predictive value (Figure 3C). Ultimately, the *F*‐test feature selection method combined with the ENetLR classifier achieved the best performance and was selected for feature screening and model development. The combined model incorporated the clinical indicators phosphate, diabetes, total Kt/V, albumin, cTnT, and GA, together with the glycated peptide biomarkers GEAFTLK(g)ATVLNYLPK and K(g)QHLFVK (Figure 3D).

Biomarker‐only and clinical‐only models were also developed, and a comparison among the three models was performed in the training set of the derivation cohort (Figure 3E; Tables 1 and S7). In the training set, the combined model achieved a ROC‐AUC of 0.784 (95% CI: 0.703–0.864), compared with 0.624 (95% CI: 0.492–0.756) for the clinical‐only model (*p* = .003).

**TABLE 1:** Performance of models.

Cohort	Train			Internal validation			External validation		
Model type	Combined	Clinical	Biomarker	Combined	Clinical	Biomarker	Combined	Clinical	Biomarker
**Specificity (95% CI)**	0.756 (0.676, 0.821)	0.779 (0.700, 0.841)	0.939 (0.884, 0.969)	0.714 (0.585, 0.816)	0.732 (0.604, 0.830)	0.911 (0.807, 0.961)	0.871 (0.783, 0.926)	0.835 (0.742, 0.899)	0.953 (0.885, 0.982)
**Sensitivity (95% CI)**	0.622 (0.461, 0.759)	0.541 (0.384, 0.690)	0.270 (0.154, 0.430)	0.750 (0.505, 0.898)	0.625 (0.386, 0.815)	0.250 (0.102, 0.495)	0.529 (0.310, 0.738)	0.529 (0.310, 0.738)	0.176 (0.062, 0.410)
**ROC AUC (95% CI)**	0.784 (0.703, 0.864)	0.624 (0.492, 0.756)	0.666 (0.552, 0.781)	0.781 (0.655, 0.907)	0.725 (0.583, 0.868)	0.612 (0.449, 0.775)	0.822 (0.718, 0.927)	0.780 (0.666, 0.894)	0.770 (0.664, 0.877)
**Average precision**	0.541	0.523	0.473	0.477	0.453	0.422	0.574	0.436	0.428
***F*1 score**	0.500	0.465	0.364	0.545	0.488	0.320	0.486	0.450	0.250
**Balanced accuracy (95% CI)**	0.689 (0.600, 0.773)	0.660 (0.571, 0.746)	0.605 (0.533, 0.680)	0.732 (0.603, 0.848)	0.679 (0.545, 0.808)	0.580 (0.478, 0.696)	0.700 (0.576, 0.824)	0.682 (0.553, 0.806)	0.565 (0.482, 0.665)
**Accuracy (95% CI)**	0.726 (0.654, 0.788)	0.726 (0.654, 0.788)	0.792 (0.724, 0.846)	0.722 (0.610, 0.812)	0.708 (0.595, 0.801)	0.764 (0.654, 0.847)	0.814 (0.727, 0.877)	0.784 (0.695, 0.853)	0.824 (0.738, 0.885)
**PPV (95% CI)**	0.418 (0.297, 0.550)	0.408 (0.282, 0.548)	0.556 (0.337, 0.754)	0.429 (0.265, 0.609)	0.400 (0.234, 0.593)	0.444 (0.189, 0.733)	0.450 (0.258, 0.658)	0.391 (0.222, 0.592)	0.429 (0.158, 0.750)
**NPV (95% CI)**	0.876 (0.803, 0.925)	0.857 (0.783, 0.909)	0.820 (0.751, 0.873)	0.909 (0.788, 0.964)	0.872 (0.748, 0.940)	0.810 (0.696, 0.888)	0.902 (0.819, 0.950)	0.899 (0.813, 0.948)	0.853 (0.768, 0.910)
**Brier score (95% CI)**	0.175 (0.146, 0.207)	0.244 (0.238, 0.251)	0.240 (0.235, 0.244)	0.186 (0.142, 0.235)	0.244 (0.241, 0.247)	0.234 (0.228, 0.240)	0.135 (0.107, 0.166)	0.241 (0.239, 0.244)	0.223 (0.216, 0.230)

*Note*: Training, *n* = 168 with 37 events; internal validation, *n* = 72 with 16 events; external validation, *n* = 102 with 17 events. The outcome was CVD within 1 year.

Abbreviations: CI, confidence interval; ROC AUC, area under the curve of receiver operating characteristic; PPV, positive predictive value; NPV, negative predictive value.

Based on the training‐set ROC analysis, the Youden‐index optimal cutoff for the combined model predicted probability was 0.373. This training‐derived cutoff was subsequently used to stratify patients into high‐risk and low‐risk groups in the internal testing set and external validation cohort (Figure 3F).

### 3.4 Model evaluation

In the internal testing set of the derivation cohort, the combined model achieved a ROC‐AUC of 0.781 (95% CI: 0.655–0.907), compared with 0.725 (95% CI: 0.583–0.868) for the clinical‐only model (Figure 4C; Tables 1 and S7).

**FIGURE 4:**
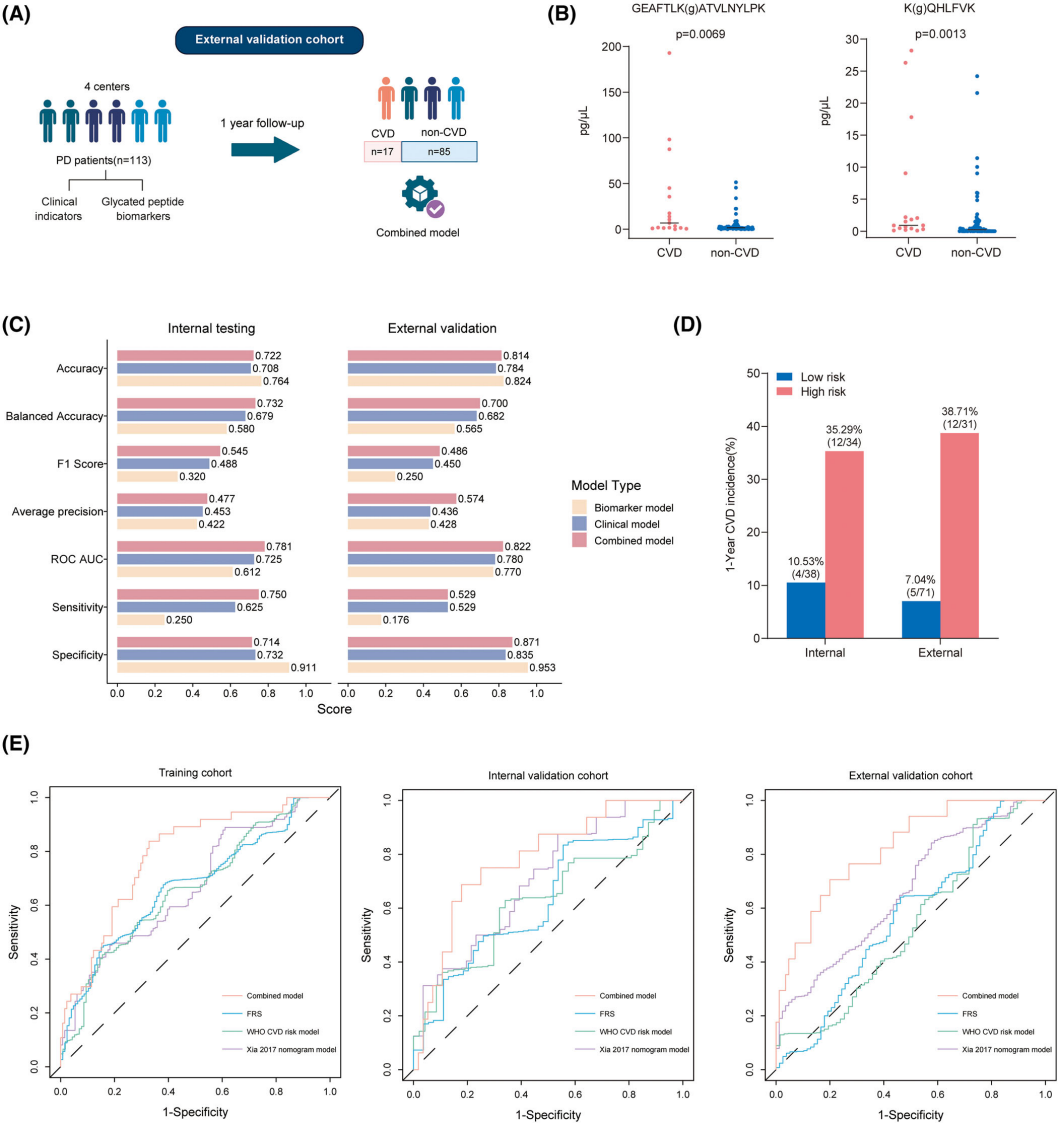
Validation of the prediction model in the testing set of the single‐centre derivation cohort and the external multicentre validation cohort. (A) The workflow of the validation phase. (B) Targeted quantification of the candidate biomarkers in the external multicentre validation cohort. (C) Comparison of model performance among the biomarker model, clinical model, and combined model in the internal testing set and the external multicentre validation cohort. (D) 1‐year CVD incidence according to risk stratification. (E) Comparison of ROC curves for the combined model and three published cardiovascular risk models.

For external validation, we prospectively enrolled 113 eligible PD patients from four other hospitals and followed them for 1 year. In total, 102 patients were included, and 17 developed CVD during follow‐up (Figure 4A). Table S8 presents the baseline characteristics of the patients in the external validation cohort. The levels of both glycated peptide biomarkers were significantly higher in patients who developed CVD than in those who did not (Figure 4B). The combined model achieved a ROC‐AUC of 0.822 (95% CI: 0.718–0.927), compared with 0.780 (95% CI: 0.666–0.894) for the clinical‐only model (Figure 4C; Tables 1 and S7).

To further evaluate its clinical applicability, the training‐derived Youden cutoff of 0.373 was used as a risk stratification threshold. Patients classified as high‐risk had substantially higher 1‐year CVD incidence than low‐risk patients in both internal and external validation cohorts (Figure 4D). Table 2 further summarises the risk stratification performance of the combined model.

**TABLE 2:** Risk stratification performance of the combined model.

	Internal validation cohort (high risk vs. low risk)	External validation cohort (high risk vs. low risk)
RR	3.35 (1.19–9.42)	5.50 (2.12–14.27)
*p* value	.021	<.001
Sensitivity, %	75.0 (47.6–92.7)	70.6 (44.0–89.7)
Specificity, %	60.7 (46.8–73.5)	77.6 (67.3–86.0)
PPV, %	35.3 (19.7–53.5)	38.7 (21.8–57.8)
NPV, %	89.5 (75.2–97.1)	93.0 (84.3–97.7)
Accuracy, %	63.9 (51.7–74.9)	76.5 (67.0–84.3)
Balanced accuracy, %	67.9 (54.9–79.5)	74.1 (61.8–85.3)

*Note*: Values are presented as estimates (95% Confidence Interval). High‐risk and low‐risk groups were defined by the training‐derived Youden cutoff of 0.373 for the combined model‐predicted probability: high‐risk, ≥0.373; low‐risk, < 0.373.

Abbreviations: RR, relative risk; PPV, positive predictive value; NPV, negative predictive value.

As supplementary reference comparisons, we evaluated the combined model against the FRS, the WHO CVD risk model, and the cardiovascular mortality nomogram developed by Xia et al. for PD patients (Table 3; Figure 4E). In the training cohort, it achieved significantly higher ROC‐AUCs than all three reference models. In external validation, it maintained significantly higher ROC‐AUCs than the FRS and WHO model, while showing a numerical advantage over the Xia model.

**TABLE 3:** Discriminative performance of the cardiovascular risk models.

Cohort	Model	ROC AUC (95% CI)	*p* value
**Training**	Combined model	0.784 (0.703, 0.864)	–
	FRS	0.669 (0.557, 0.781)	.021
	WHO CVD risk model	0.659 (0.552, 0.766)	.026
	Xia 2017 nomogram model	0.665 (0.564, 0.765)	.026
**Internal validation**	Combined model	0.781 (0.655, 0.907)	–
	FRS	0.629 (0.452, 0.805)	.099
	WHO CVD risk model	0.630 (0.444, 0.816)	.190
	Xia 2017 nomogram model	0.706 (0.564, 0.849)	.239
**External validation**	Combined model	0.822 (0.718, 0.927)	–
	FRS	0.567 (0.414, 0.719)	<.001
	WHO CVD risk model	0.536 (0.374, 0.697)	<.001
	Xia 2017 nomogram model	0.662 (0.517, 0.808)	.082

*Note*: The combined model refers to the model constructed in our study. The FRS refers to the 2008 lipid‐based model; the WHO CVD risk model refers to the 2019 laboratory‐based model for East Asia; and the Xia 2017 nomogram model refers to the cardiovascular mortality prediction nomogram for peritoneal dialysis patients developed by Xia et al. in 2017. Paired AUC comparisons used DeLong covariance estimates.

Abbreviations: ROC, receiver operating characteristic; AUC, area under the curve; CI, confidence interval; FRS, Framingham risk score; WHO, World Health Organization; CVD, cardiovascular disease.

### 3.5 Online CVD risk calculator for PD patients

We developed a web application based on our combined model with eight variables and deployed it online, enabling clinicians to conveniently assess the risk of CVD in PD patients. The model integrates six clinical indicators with two glycated peptide biomarkers to provide individualised cardiovascular risk prediction. The website is available at https://glycapeppd‐cvd‐risk‐calculator.streamlit.app.

## 4 DISCUSSION

Characterising early glycated proteomic features associated with CVD in PD patients, identifying potential biomarkers, and developing a PD‐specific prediction model may facilitate early recognition of CVD and enable timely intervention. Such glycated peptide biomarkers may also help elucidate the molecular mechanisms underlying CVD in PD and serve as potential therapeutic targets. In this study, we combined glycated peptide enrichment, high‐throughput proteomics, and ML analyses to identify two novel glycated peptide biomarkers. We then integrated these biomarkers with clinical indicators to develop a CVD prediction model for PD patients and validated its predictive value in both internal and external cohorts.

Proteomics enables systematic profiling of the proteome and its dynamic alterations in biological systems, providing a powerful approach to elucidate disease pathogenesis and creating new opportunities for early disease detection. Notably, protein glycation, a pathological modification, can induce substantial alterations in protein structure and function. Glycation refers to the non‐enzymatic covalent attachment of sugars or sugar derivatives to proteins and is generally divided into two stages: the formation of early‐stage EGPs and the generation of late‐stage AGEs.^13^, ^24^, ^25^ Accumulating evidence has linked AGEs to CVD in dialysis patients.^26^, ^27^, ^28^ However, most studies have focused on HD patients, and data in PD patients remain limited. Moreover, as late‐stage end products, AGEs primarily reflect the cumulative effects of long‐term metabolic dysregulation and oxidative stress rather than early pathological changes, thereby limiting the sensitivity for early risk prediction.^29^, ^30^ In addition, the lack of standardised quantification methods further restricts their clinical applicability.^31^, ^32^ Consequently, the utility of AGEs for predicting CVD in PD patients remains limited. Importantly, PD patients are exposed to a high glucose load from conventional glucose‐based dialysate, which may increase the overall glycation burden.^15^ Therefore, comprehensive characterisation of the glycated proteomics in PD patients is warranted. In our study, we performed high‐throughput profiling and analysis of early‐stage glycated proteomics in PD patients. Because EGPs arise at the initial stage of glycation, they may reflect short‐term glucose exposure and metabolic perturbations more sensitively, making them better suited for early risk assessment. Although GA and HbA1c have been associated with CVD in dialysis patients, they are generally reported in clinical practice as relative glycation indices rather than absolute concentrations of specific glycated products. In contrast, by using synthetic heavy isotope‐labelled standards and PRM‐based LC‐MS/MS, we established a targeted absolute quantification method for glycated peptide biomarkers, enabling more accurate and reproducible measurement.

In the prospective derivation cohort, we performed targeted quantification of the four candidate biomarkers and ultimately prioritised two glycated peptides, GEAFTLK(g)ATVLNYLPK and K(g)QHLFVK, derived from α2M and ApoB, respectively. ApoB is the primary protein component of very low‐density lipoproteins, intermediate‐density lipoprotein, and LDL particles.^33^ Mechanistically, ApoB‐100 mediates proteoglycan binding and arterial retention of LDL which is an initiating step in atherogenesis.^34^, ^35^ In peritoneal dialysis populations, higher ApoB levels have been associated with greater plaque burden, vascular events, and cardiovascular mortality.^36^, ^37^, ^38^ Studies have shown that glycated ApoB might promote LDL retention and increase CVD risk.^39^, ^40^, ^41^ The glycated peptide K(g)QHLFVK contains a glycated lysine at K1476, located within the classical β1 lipid‐binding region of ApoB. Recent studies further showed that ApoB forms an extended amphipathic β‐sheet belt on the LDL surface, contributing to the shape and structural cohesion of LDL.^42^, ^43^ K1476 lies within a glycation‐sensitive ApoB epitope that overlaps with an AGE‐reactive region identified in earlier studies.^44^, ^45^ These findings suggest that glycation at K1476 may alter the local conformation of ApoB, thereby indirectly affecting LDL receptor‐mediated lipoprotein clearance.

As for α2M, it was an abundant circulating protease inhibitor and acute‐phase protein. Previous targeted proteomics studies have shown that α2M could identify coronary heart disease in diabetic patients with high accuracy, supporting its potential as a CVD biomarker.^46^, ^47^ Mechanistically, activated α2M signals via LRP1 to modulate PI3K/Akt–ERK pathways, attenuate lipid‐induced cellular injury, and influence insulin responses, supporting a link to atherogenesis and cardiometabolic injury.^48^ Although the biological roles of the glycated peptide remain unclear, its potential function may be inferred from the structural context of the glycation sites. The peptide GEAFTLK(g)ATVLNYLPK contains a glycated lysine at K811, located near a hinge region involved in the conformational rearrangement of the protein. Proteolytic cleavage of the bait region triggers large‐scale structural changes that enable protease entrapment and exposure of functional domains.^49^ Notably, K811 has also been reported as an acetylation site, suggesting that this residue is accessible to modification.^50^ Therefore, glycation at K811 may alter local charge and steric properties, potentially affecting conformational dynamics and protease‐regulatory function.

Several well‐established CVD risk prediction models are currently available. The FRS and WHO CVD risk models provide practical frameworks for cardiovascular risk assessment using routinely available clinical variables. However, their 10‐year primary‐prevention focus and omission of dialysis‐related predictors limit their direct applicability to short‐term CVD prediction in PD patients.^21^, ^22^ The nomogram developed by Xia et al. addresses several characteristics relevant to PD patients by incorporating CVD history, serum albumin, haemoglobin, C‐reactive protein, and urine output. Nevertheless, its original prediction target is cardiovascular mortality at 1 and 3 years, which differs from the broader outcome of new or recurrent CVD events evaluated in our study.^23^ This distinction leaves a clinical need for models specifically developed for short‐term prediction of CVD in PD patients. By selecting PD‐specific biomarkers and clinical indicators, our model provided a more targeted and precise tool for CVD prediction in PD patients. When integrated with clinical indicators, proteomics can facilitate the development of highly accurate CVD risk prediction models.^51^, ^52^ An increasing number of ML‐based models have been developed to predict CVD risk in PD patients,^53^, ^54^, ^55^ but most rely on clinical indicators, lack specific biomarkers, and have undergone limited external validation and model benchmarking. In our study, we integrated specific biomarkers with clinical indicators and applied multiple ML models for model development and validation across internal and external cohorts. This process ultimately generated a prediction model built on phosphate, diabetes, total Kt/V, albumin, cTnT, GA, GEAFTLK(g)ATVLNYLPK, and K(g)QHLFVK.

Hyperphosphataemia has been recognised as a predictor of CVD in PD patients. Excess phosphate promotes vascular calcification by inducing osteogenic changes in vascular smooth muscle cells and increasing fibroblast growth factor 23 and iPTH.^56^, ^57^ Clinically, higher serum phosphate levels have been associated with increased cardiovascular mortality and have been shown to predict the progression of coronary artery calcification in PD cohorts.^58^, ^59^ In parallel, diabetes mellitus and CKD are established risk factors for CVD, and their coexistence further increases this risk.^60^, ^61^ Moreover, the glucose exposure from peritoneal dialysate may exacerbate dysglycaemia.^62^ GA, a marker of short‐term glycaemic control, has been considered more suitable than HbA1c for assessing glycaemic status in PD patients, because it is less affected by anaemia and iron therapy. GA has also been linked to subclinical atherosclerosis and adverse cardiovascular outcomes in HD patients.^63^, ^64^, ^65^ However, given the high prevalence of hypoalbuminaemia in PD patients, concerns have been raised regarding the applicability of GA in this population. Importantly, albumin has been consistently associated with cardiovascular risk and prognosis, and reflects nutritional and inflammatory status. In PD cohorts, lower albumin levels have been shown to predict CVD readmission and were associated with higher cardiovascular mortality.^66^, ^67^, ^68^ Therefore, our model incorporated diabetes, GA, and albumin and explicitly accounted for their interactions, thereby partially addressing this limitation. cTnT is a key regulatory protein involved in myocardial contraction and has been widely used for CVD diagnosis and risk stratification in the general population. It is also closely associated with CVD in dialysis patients. Both Chen et al. and Han et al. reported that cTnT was a risk factor for CVD in PD patients.^69^, ^70^ Although not a marker directly related to CVD, total Kt/V is a key indicator of dialysis adequacy and the clearances of small uremic solutes, and it has also been recognised as a prognostic predictor in PD patients. Liu et al. reported that patients with adequate dialysis had significantly better clinical outcomes than those with inadequate dialysis.^71^ In addition, another study has shown that Kt/V was positively correlated with IMT, an early marker of atherosclerosis, suggesting that dialysis adequacy may be a potential contributor to the atherosclerotic process.^72^ Overall, the biomarkers and clinical indicators included in our model were selected based on rigorous ML analyses, established clinical relevance and supporting evidence from prior studies, yielding a scientifically predictive model for CVD risk estimation.

In addition to evaluating discrimination, we used the training‐derived Youden cutoff of 0.373 to stratify patients into high‐risk and low‐risk groups. Patients classified as high‐risk had a significantly higher incidence of CVD than those classified as low‐risk, demonstrating the clinical relevance of the model for risk stratification. The negative predictive values of 89.5% and 93.0% in the internal and external validation cohorts, respectively, further support its potential to identify patients at relatively lower 1‐year CVD risk. These findings support its potential for guiding intensified surveillance and earlier intervention in high‐risk PD patients.

However, our study had several limitations. First, although we identified glycated peptide biomarkers specific to PD patients, mechanistic evidence from basic research was lacking, and their pathogenic roles remain to be further elucidated. Second, although the combined model was validated in both internal and external cohorts, the relatively small cohort and limited number of outcome events constrain the precision of performance estimates and the assessment of model generalisability. Further validation in larger, geographically diverse, multicentre cohorts is therefore needed. Third, reliance on PRM‐LC‐MS/MS may limit clinical applicability because of its relatively high cost and technical requirements. Future studies should focus on developing simplified assays and dedicated detection kits. Also, excluding participants after enrolment, including those who died of non‐cardiovascular causes, may have introduced bias and limit further assessment of its impact on the combined model.

## 5 CONCLUSION

In summary, our study characterised the glycated proteomics associated with CVD in PD patients, identified glycated peptide biomarkers with predictive value using ML approaches, and developed a CVD prediction model by integrating the biomarkers with clinical indicators. The model's performance was validated in both internal and external cohorts, providing a basis for earlier cardiovascular risk stratification and targeted preventive interventions in PD patients.

## AUTHOR CONTRIBUTIONS

Ji Ji, Haojie Lu, and Xiaofang Yu designed the study. Ji Ji, Zhaoying Nie, Na Jiang, Ting Xie, Lei Zhang, Xiaoyan Liu, Min Lu, Bo Xiang, and Weiwei Wu collected the samples of the patients. Ji Ji, Zhaoying Nie, Na Jiang, Ting Xie, and Lei Zhang collected the data and did the investigation. Ji Ji and Zhaoying Nie did the formal analysis. Xiaotian Jiang, Lin Zhang, Jun Ji, Jianzhou Zou, Xiaoqiang Ding, Haojie Lu, and Xiaofang Yu supervised the project. Zhaoying Nie and Min Lu performed the validation. Ji Ji wrote the original draft. Haojie Lu and Xiaofang Yu reviewed and edited the article and were responsible for the funding acquisition and project administration.

## CONFLICT OF INTEREST STATEMENT

The authors declare no conflicts of interest.

## ETHICS STATEMENT

This study was conducted in accordance with the principles of the Declaration of Helsinki and was approved by the Ethical Committee of Zhongshan Hospital, Fudan University (approval No. B2017‐108R and B2022‐161R). Written informed consent was obtained from all participants. All data collection, storage, and analysis procedures were performed in accordance with relevant ethical guidelines to protect participants’ privacy and confidentiality.

[1] Ma J, Chen T, Wu S, et al. iProX: an integrated proteome resource. Nucleic Acids Res. 2019;47(D1):D1211–D1217.

[2] Chen T, Ma J, Liu Y, et al. iProX in 2021: connecting proteomics data sharing with big data. Nucleic Acids Res. 2022;50(D1):D1522–D1527.

## Supporting information



Supporting Information

## Data Availability

The data underlying this article will be shared on reasonable request to the corresponding author. The mass spectrometry proteomics data have been deposited to the ProteomeXchange Consortium (https://proteomecentral.proteomexchange.org) via the iProX partner repository [1,2] with the dataset identifiers PXD076856 and PXD076654.
