# Supplementary material for: Predicting cardiovascular disease in peritoneal dialysis patients using glycated proteomics and machine learning: A prospective multicentre study
Source: Clin Transl Med. 2026 Oct 27;16(11):e70889. doi: 10.1002/ctm2.70889 (PMC13714096; doi:10.1002/ctm2.70889)
Supplement: Supplementary file 1 [file CTM2-16-e70889-s001.docx]

**Supplemental material**

Table S1. Precursor-product ion transitions selected for PRM analysis of target glycated peptides

Table S2. Characteristics of the discovery cohort

Table S3. Performance of machine learning models in the discovery cohort

Table S4. Glycated peptides selected using glmnet model

Table S5. Detectability of glycated peptides and their associations with CVD

Table S6. Characteristics of the internal derivation cohort

Table S7. Pairwise comparisons of the combined, clinical, and biomarker models

Table S8. Characteristics of the multicenter external validation cohort

Table S9. Number and reasons for participant dropout

Table S10. Composition of cardiovascular disease events in three cohorts

Table S11. Predictive values and Brier scores of the three models in validation cohorts

Table S12. Covariate-adjusted associations per interquartile-range increase of glycated peptide with 1-year CVD in the derivation cohort

Figure S1. ROC curves of the four candidate glycated peptide biomarkers individually and in combination

Figure S2. Calibration of the combined, clinical-only, and biomarker-only models in the internal and external validation cohorts

Figure S3. Decision curve analysis of three models in the validation cohorts

**Table S1. Precursor-product ion transitions selected for PRM analysis of target glycated peptides**

| **Protein/Peptide** | **Label type** | **Precursor (Q1)** | **Charge state (Q1)** | **Ion** | **Product (Q3)** | **Charge state (Q3)** |
| --- | --- | --- | --- | --- | --- | --- |
| Alpha-1B-glycoprotein/  SGLSTGWTQLSK(g)LLELTGPK | H | 763.4206 | 3 | b3 | 258.1448 | 1 |
|  |  |  |  | y3 | 309.2012 | 1 |
|  |  |  |  | y2 | 252.1797 | 1 |
|  | L | 760.7492 | 3 | y3 | 301.1870 | 1 |
|  |  |  |  | y2 | 244.1655 | 1 |
|  |  |  |  | b3 | 258.1448 | 1 |
| Alpha-1-antitrypsin/  ELDRDTVFALVNYIFFK(g)GK | H | 612.5791 | 4 | y6 | 911.5327 | 1 |
|  |  |  |  | y5 | 798.4487 | 1 |
|  |  |  |  | b7 | 829.4050 | 1 |
|  | L | 610.5756 | 4 | y5 | 790.4345 | 1 |
|  |  |  |  | b7 | 829.4050 | 1 |
|  |  |  |  | b6 | 730.3366 | 1 |
| Alpha-2-macroglobulin/  GEAFTLK(g)ATVLNYLPK | H | 646.3622 | 3 | y6 | 755.4541 | 1 |
|  |  |  |  | y7 | 854.5225 | 1 |
|  |  |  |  | y8 | 955.5702 | 1 |
|  | L | 643.6908 | 3 | y6 | 747.4399 | 1 |
|  |  |  |  | y7 | 846.5083 | 1 |
|  |  |  |  | y8 | 947.5560 | 1 |
| Apolipoprotein B-100/  K(g)QHLFVK | H | 357.8811 | 3 | y3 | 401.2638 | 1 |
|  |  |  |  | y2 | 254.1954 | 1 |
|  |  |  |  | y1 | 155.1270 | 1 |
|  | L | 355.2097 | 3 | y3 | 393.2496 | 1 |
|  |  |  |  | y2 | 246.1812 | 1 |
|  |  |  |  | y1 | 147.1128 | 1 |

**Table S2. Characteristics of the discovery cohort**

| Characteristic | CVD (n=48) | Non-CVD (n=48) | P value |
| --- | --- | --- | --- |
| Age, y | 64 (33-80) | 64 (35-80) | 0.921 |
| Gender, male | 24，50.0% | 24，50.0% | - |
| Body mass index, kg/m^2^ | 24.9 (13.2-41.6) | 25.4 (17.4-32.0) | 0.488 |
| Dialysis vintage, m | 34 (6-144) | 34 (8-150) | 0.982 |
| Hypertension, % | 47, 97.9% | 48, 100% | 1.000 |
| Diabetes, % | 18, 37.5% | 13, 27.1% | 0.383 |
| Antiplatelet drugs, % | 18, 37.5% | 7, 14.6% | 0.019 |
| Anemia drugs, % | 45, 93.8% | 40, 83.3% | 0.199 |
| Lipid-lowering drugs, % | 26, 54.2% | 18, 37.5% | 0.151 |
| Phosphate binders, % | 16, 33.3% | 31, 64.6% | 0.004 |
| SBP, mmHg | 139 (74-195) | 143 (100-198) | 0.623 |
| DBP, mmHg | 79 (52-120) | 82 (54-106) | 0.299 |
| Hemoglobin, g/L | 98 (50-128) | 106 (77-139) | 0.060 |
| Albumin, g/L | 32 (17-59) | 35 (22-43) | 0.026 |
| FPG, mmol/L | 6.1 (3.3-16.0) | 5.6 (2.9-13.5) | 0.045 |
| HbA1c, % | 6.3 (4.8-9.3) | 5.8 (4.4-10.6) | 0.009 |
| GA, % | 15.0 (8.0-27.3) | 14.6 (8.0-44.0) | 0.420 |
| Scr, µmol/L | 884 (303-1298) | 930 (224-1944) | 0.257 |
| BUN, mmol/L | 18.1 (9.0-26.7) | 20.1 (8.1-44.7) | 0.288 |
| eGFR, mL/min/1.73m^2^ | 5.14 (2.58-22.20) | 4.79 (1.89-22.36) | 0.298 |
| TC, mmol/L | 3.97 (1.87-6.27) | 4.22 (2.18-6.60) | 0.406 |
| Triglyceride, mmol/L | 1.80 (0.16-4.66) | 1.94 (0.63-9.10) | 0.926 |
| LDL-C, mmol/L | 2.23 (0.54-4.13) | 2.37 (0.94-4.03) | 0.452 |
| HDL-C, mmol/L | 1.06 (0.37-3.77) | 1.11 (0.51-5.31) | 0.716 |
| CRP, mg/L | 18.5 (0.3-161.4) | 12.6 (0.3-189.7) | 0.096 |
| NT-proBNP, pg/mL | 17518.5 (371.4-35000.0) | 7930.4 (506.0-35000.0) | ＜0.001 |
| cTnT, ng/mL | 0.196 (0.030-1.360) | 0.099 (0.005-0.890) | ＜0.001 |
| 24-h urine volume, mL | 350 (0-2650) | 440 (0-2000) | 0.244 |
| Peritoneal Kt/V | 1.67 (0.47-3.00) | 1.58 (0.59-4.00) | 0.490 |
| Renal Kt/V | 0.25 (0-1.38) | 0.32 (0-1.51) | 0.526 |
| Total Kt/V | 1.92 (1.16-3.40) | 1.86 (1.00-4.00) | 0.447 |
| Peritoneal Ccr, L/week/1.73m^2^ | 45.69 (17.01-73.61) | 45.50 (16.90-104.01) | 0.940 |
| Renal Ccr, L/week/1.73m^2^ | 14.52 (0-75.46) | 15.41 (0-71.16) | 0.813 |
| Total Ccr, L/week/1.73m^2^ | 58.40 (35.63-107.89) | 60.91 (33.98-104.04) | 0.585 |

Values are presented as means ± standard deviation or medians (range) for continuous variables and count (percentage) for categorical variables. eGFR indicates estimated glomerular filtration rate; DBP, diastolic blood pressure; SBP, systolic blood pressure; FPG, fasting plasma glucose; HbA1c, glycated hemoglobin; GA, glycated albumin; Scr, serum creatinine; BUN, blood urea nitrogen; TC, total cholesterol; LDL-C, low-density lipoprotein cholesterol; HDL-C, high-density lipoprotein cholesterol; CRP, C-reactive protein; NT-proBNP, N-terminal pro-brain natriuretic peptide; cTnT, cardiac troponin T; Ccr, creatinine clearance rate.

**Table S3. Performance of machine learning models in the discovery cohort**

| **Model** | **ROC-AUC** | **Accuracy** | **F1-score** |
| --- | --- | --- | --- |
| Generalized linear model network | 0.923 ± 0.066 | 0.875 ± 0.061 | 0.879 ± 0.041 |
| Generalized linear model boosting | 0.899 ± 0.091 | 0.843 ± 0.084 | 0.849 ± 0.059 |
| Support vector machine | 0.894 ± 0.074 | 0.760 ± 0.072 | 0.762 ± 0.067 |
| Light gradient boosting machine | 0.892 ± 0.056 | 0.781 ± 0.102 | 0.766 ± 0.124 |
| Random forest | 0.888 ± 0.092 | 0.730 ± 0.104 | 0.730 ± 0.127 |
| Gaussian process classifier | 0.888 ± 0.106 | 0.739 ± 0.065 | 0.718 ± 0.071 |
| Gradient boosting machine | 0.882 ± 0.066 | 0.792 ± 0.050 | 0.798 ± 0.061 |
| Generalized additive model boosting | 0.871 ± 0.096 | 0.771 ± 0.079 | 0.777 ± 0.083 |
| Multivariate adaptive regression splines | 0.849 ± 0.070 | 0.812 ± 0.081 | 0.811 ± 0.111 |
| k-nearest neighbors | 0.834 ± 0.043 | 0.655 ± 0.083 | 0.579 ± 0.081 |
| Extreme gradient boosting | 0.834 ± 0.106 | 0.781 ± 0.127 | 0.776 ± 0.165 |
| Neural network | 0.794 ± 0.084 | 0.792 ± 0.050 | 0.815 ± 0.052 |
| Naive Bayes | 0.793 ± 0.125 | 0.718 ± 0.116 | 0.706 ± 0.102 |
| Linear discriminant analysis | 0.768 ± 0.123 | 0.707 ± 0.129 | 0.695 ± 0.121 |
| Feedforward neural network | 0.674 ± 0.084 | 0.676 ± 0.089 | 0.669 ± 0.051 |
| Logistic regression | 0.624 ± 0.151 | 0.646 ± 0.112 | 0.631 ± 0.175 |

**Table S4. Glycated peptides selected using glmnet model**

| **No.** | **Glycated peptide sequence** | **UniProt accession** | **Corresponding protein** |
| --- | --- | --- | --- |
| 1 | SGLSTGWTQLSK(+164.07)LLELTGPK | [P04217](https://www.uniprot.org/uniprotkb/P04217/entry) | Alpha-1B-glycoprotein |
| 2 | RQGALELIK(+164.07)K | [P01024](https://www.uniprot.org/uniprotkb/P01024/entry) | Complement C3 |
| 3 | IFTVNHK(+164.07)LLPVGR | [P01024](https://www.uniprot.org/uniprotkb/P01024/entry) | Complement C3 |
| 4 | AK(+164.07)DQLTCNKFDLK | [P01024](https://www.uniprot.org/uniprotkb/P01024/entry) | Complement C3 |
| 5 | EQLK(+164.07)AVMDDFAAFVEK | [P02768](https://www.uniprot.org/uniprotkb/P02768/entry) | Albumin |
| 6 | AK(+164.07)RMPCAEDYLSVVLNQLCVLHEK | [P02768](https://www.uniprot.org/uniprotkb/P02768/entry) | Albumin |
| 7 | AVMDDFAAFVEK(+164.07)CCK | [P02768](https://www.uniprot.org/uniprotkb/P02768/entry) | Albumin |
| 8 | FKDLGEENFK(+164.07)ALVLIAFAQYLQQCPFEDHVK | [P02768](https://www.uniprot.org/uniprotkb/P02768/entry) | Albumin |
| 9 | DAHK(+164.07)SEVAHRFK | [P02768](https://www.uniprot.org/uniprotkb/P02768/entry) | Albumin |
| 10 | ETYGEMADCCAK(+164.07)QEPER | [P02768](https://www.uniprot.org/uniprotkb/P02768/entry) | Albumin |
| 11 | APELLFFAK(+164.07)R | [P02768](https://www.uniprot.org/uniprotkb/P02768/entry) | Albumin |
| 12 | K(+164.07)QTALVELVKHKPK | [P02768](https://www.uniprot.org/uniprotkb/P02768/entry) | Albumin |
| 13 | MPCAEDYLSVVLNQLCVLHEK(+164.07)TPVSDR | [P02768](https://www.uniprot.org/uniprotkb/P02768/entry) | Albumin |
| 14 | SHCIAEVENDEMPADLPSLAADFVESK(+164.07)DVCK(+164.07)NYAEAKDVFL | [P02768](https://www.uniprot.org/uniprotkb/P02768/entry) | Albumin |
| 15 | QNCELFEQLGEYK(+164.07)FQNALLVR | [P02768](https://www.uniprot.org/uniprotkb/P02768/entry) | Albumin |
| 16 | KK(+164.07)YLYEIAR | [P02768](https://www.uniprot.org/uniprotkb/P02768/entry) | Albumin |
| 17 | CTAFHDNEETFLK(+164.07)KYLYEIAR | [P02768](https://www.uniprot.org/uniprotkb/P02768/entry) | Albumin |
| 18 | ADDKETCFAEEGKK(+164.07) | [P02768](https://www.uniprot.org/uniprotkb/P02768/entry) | Albumin |
| 19 | QIK(+164.07)K(+164.07)QTALVELVK | [P02768](https://www.uniprot.org/uniprotkb/P02768/entry) | Albumin |
| 20 | SPLFMGK(+164.07)VVNPTQK | [P01009](https://www.uniprot.org/uniprotkb/P01009/entry) | Alpha-1-antitrypsin |
| 21 | ELDRDTVFALVNYIFFK(+164.07)G | [P01009](https://www.uniprot.org/uniprotkb/P01009/entry) | Alpha-1-antitrypsin |
| 22 | FNKPFVFLMIEQNTK(+164.07)SPLFMGK | [P01009](https://www.uniprot.org/uniprotkb/P01009/entry) | Alpha-1-antitrypsin |
| 23 | ELDRDTVFALVNYIFFK(+164.07)GK | [P01009](https://www.uniprot.org/uniprotkb/P01009/entry) | Alpha-1-antitrypsin |
| 24 | SVPMVPPGIK(+164.07)YLYLR | [P51884](https://www.uniprot.org/uniprotkb/P51884/entry) | Lumican |
| 25 | MK(+164.07)YWGVASFLQ | [P02753](https://www.uniprot.org/uniprotkb/P02753/entry) | Retinol-binding protein 4 |
| 26 | MK(+164.07)YWGVASFLQK | [P02753](https://www.uniprot.org/uniprotkb/P02753/entry) | Retinol-binding protein 4 |
| 27 | GYILVGQAK(+164.07)LSCSYSHWSAPAPQCK | [P04003](https://www.uniprot.org/uniprotkb/P04003/entry) | C4b-binding protein alpha chain |
| 28 | GEAFTLK(+164.07)ATVLNYLPK | [P01023](https://www.uniprot.org/uniprotkb/P01023/entry) | Alpha-2-macroglobulin |
| 29 | AVPK(+164.07)SSSNEEVMFLTVQVK | [P01023](https://www.uniprot.org/uniprotkb/P01023/entry) | Alpha-2-macroglobulin |
| 30 | QLEGGLK(+164.07)QFSFPLSSEPFQGSYK | [P01023](https://www.uniprot.org/uniprotkb/P01023/entry) | Alpha-2-macroglobulin |
| 31 | K(+164.07)QHLFVK | [P04114](https://www.uniprot.org/uniprotkb/P04114/entry) | Apolipoprotein B-100 |
| 32 | SIDVACHPGYALPK(+164.07)AQTTVTCMENGWSPTPR | [P08603](https://www.uniprot.org/uniprotkb/P08603/entry) | Complement factor H |
| 33 | LK(+164.07)LLDNWDSVTSTFSK | [P02647](https://www.uniprot.org/uniprotkb/P02647/entry) | Apolipoprotein A-I |
| 34 | LQAILGVPWK(+164.07)DKNCTSR | [P01019](https://www.uniprot.org/uniprotkb/P01019/entry) | Angiotensinogen |
| 35 | CLVNLIEK(+164.07)VASYGVKPR | [P00751](https://www.uniprot.org/uniprotkb/P00751/entry) | Complement factor B |
| 36 | YGLVTYATYPK(+164.07)IWVK | [P00751](https://www.uniprot.org/uniprotkb/P00751/entry) | Complement factor B |
| 37 | AFIQLWAFDAVK(+164.07)GK | [P02760](https://www.uniprot.org/uniprotkb/P02760/entry) | Protein AMBP |
| 38 | YAFELK(+164.07)EK | [P02748](https://www.uniprot.org/uniprotkb/P02748/entry) | Complement component C9 |
| 39 | TAGWNIPMGLLYNK(+164.07)INHCR | [P02787](https://www.uniprot.org/uniprotkb/P02787/entry) | Serotransferrin |
| 40 | SVIPSDGPSVACVK(+164.07)KASYLDCIR | [P02787](https://www.uniprot.org/uniprotkb/P02787/entry) | Serotransferrin |
| 41 | EFQLFSSPHGK(+164.07)DLLFK | [P02787](https://www.uniprot.org/uniprotkb/P02787/entry) | Serotransferrin |
| 42 | LYLVQGTQVYVFLTK(+164.07)GGYTLVSGYPK | [P02790](https://www.uniprot.org/uniprotkb/P02790/entry) | Hemopexin |
| 43 | APK(+164.07)LLIYDASNLETGVPSR | [P01593](https://www.uniprot.org/uniprotkb/P01593/entry) | Immunoglobulin kappa variable 1D-33 |
| 44 | VNHVTLSQPKIVK(+164.07) | [P61769](https://www.uniprot.org/uniprotkb/P61769/entry) | Beta-2-microglobulin |
| 45 | K(+164.07)FPSGTFEQVSQLVK | [P02774](https://www.uniprot.org/uniprotkb/P02774/entry) | Vitamin D-binding protein |
| 46 | YARWFQQK(+164.07)PGQAPVLVIYK | [P01718](https://www.uniprot.org/uniprotkb/P01718/entry) | Immunoglobulin lambda variable 3-27 |
| 47 | CCVECPPCPAPPVAGPSVFLFPPK(+164.07)PKDTLMISR | [P01859](https://www.uniprot.org/uniprotkb/P01859/entry) | Immunoglobulin heavy constant gamma 2 |
| 48 | TPK(+164.07)SPVGVQPILNEHTFCAGMSK | [P00738](https://www.uniprot.org/uniprotkb/P00738/entry) | Haptoglobin |
| 49 | TEGDGVYTLNNEK(+164.07)QWINK | [P00738](https://www.uniprot.org/uniprotkb/P00738/entry) | Haptoglobin |
| 50 | K(+164.07)FICPLTGLWPINTLK | [P02749](https://www.uniprot.org/uniprotkb/P02749/entry) | Beta-2-glycoprotein 1 |
| 51 | FSYSSGHVHLSSENK(+164.07)FQNSAILTIQPK | [P01031](https://www.uniprot.org/uniprotkb/P01031/entry) | Complement C5 |
| 52 | AAVYQPQPHPQPPPYGHCVTDSGVVYSVGMQWLK(+164.07)TQGNK | [P02751](https://www.uniprot.org/uniprotkb/P02751/entry) | Fibronectin |
| 53 | SK(+164.07)FTCACPDQFK | [Q14520](https://www.uniprot.org/uniprotkb/Q14520/entry) | Factor VII-activating protease |
| 54 | ASQGISNYLAWYQQK(+164.07)PGK | [A0A075B6S5](https://www.uniprot.org/uniprotkb/A0A075B6S5/entry) | Immunoglobulin kappa variable 1-27 |
| 55 | SCDK(+164.07)THTCPPCPAPELLGGPSVF | [P0DOX5](https://www.uniprot.org/uniprotkb/P0DOX5/entry) | Immunoglobulin gamma-1 heavy chain |

Glycated site is labeled as (+164.07) at the corresponding position.

**Table S5. Detectability of glycated peptides and their associations with CVD**

| **Glycated peptide** | **Below LOD**  **n, %** | **ρ**  **(95% CI)** | **P value** |
| --- | --- | --- | --- |
| GEAFTLK(g)ATVLNYLPK (alpha-2-macroglobulin) | 15, 4.4% | 0.233 (0.129,0.329) | ＜0.001 |
| K(g)QHLFVK  (apolipoprotein B-100) | 11, 3.2% | 0.225 (0.121,0.323) | ＜0.001 |
| SGLSTGWTQLSK(g)LLELTGPK (alpha-1β-glycoprotein) | 168, 49.1% | 0.018 (-0.077,0.114) | 0.739 |
| ELDRDTVFALVNYIFFK(g)GK (alpha-1-antitrypsin) | 32, 9.4% | 0.128 (0.021,0.230) | 0.036 |

Abbreviations: CI, confidence interval; CVD, cardiovascular disease; LOD, limit of detection.

**Table S6. Characteristics of the internal derivation cohort**

| Characteristic | Overall (n=240) | Presence of CVD (n=53) | Absence of CVD (n=187) | P value |
| --- | --- | --- | --- | --- |
| Age, y | 56 (24-80) | 60 (40-80) | 55 (24-80) | 0.0025 |
| Gender, male | 139 , 57.9% | 34, 64.2% | 105, 56.1% | 0.3456 |
| Body mass index, kg/m^2^ | 25.8 (16.0-34.8) | 24.7 (18.1-33.9) | 26.1 (16.0-34.8) | 0.1441 |
| Dialysis vintage, m | 42 (7-182) | 49 (8-138) | 40 (7-182) | 0.6612 |
| Hypertension, % | 228, 95.0% | 52, 98.1% | 176, 94.1% | 0.4729 |
| Diabetes, % | 51, 21.3% | 25, 47.2% | 26, 13.9% | ＜0.001 |
| History of CVD, % | 96, 40.0% | 23, 43.4% | 73, 39.0% | 0.6344 |
| Smoking, % | 28, 11.7% | 3, 5.7% | 25, 13.4% | 0.1498 |
| Use of antihypertensive drugs,% | 228, 95.0% | 52, 98.1% | 176, 94.1% | 0.4729 |
| Use of glucose-lowering drugs, % | 51, 21.3% | 25, 47.2% | 26, 13.9% | ＜0.001 |
| Use of phosphate binders, % | 95, 39.6% | 27, 50.9% | 68, 36.4% | 0.0585 |
| SBP, mmHg | 136 (78-208) | 142 (78-208) | 134 (82-200) | 0.0046 |
| DBP, mmHg | 83 (54-115) | 81 (54-108) | 83 (57-115) | 0.4401 |
| RBC, ×10^12^/L | 3.49 (1.48-5.62) | 3.41 (1.56-5.44) | 3.51 (1.48-5.62) | 0.3717 |
| Hemoglobin, g/L | 106 (44-157) | 102 (44-152) | 107 (56-157) | 0.1063 |
| Platelet, ×10^9^/L | 196 (40-424) | 189 (72-313) | 198 (40-424) | 0.3962 |
| WBC, ×10^9^/L | 6.33 (1.52-13.97) | 6.51 (1.52-13.97) | 6.28 (2.14-13.14) | 0.8886 |
| NEUT, % | 67.2 (36.2-91.2) | 68.4 (48.3-91.2) | 66.9 (36.2-84.8) | 0.7477 |
| TB, μmol/L | 5.2 (1.5-35.3) | 5.1 (1.5-35.3) | 5.2 (1.8-28.7) | 0.0194 |
| DBIL, μmol/L | 1.5 (0.1-21.1) | 1.7 (0.2-21.1) | 1.4 (0.1-10.2) | 0.2229 |
| TP, g/L | 60 (28-85) | 59 (43-73) | 61 (28-85) | 0.0397 |
| Albumin, g/L | 35 (18-48) | 33 (22-43) | 35 (18-48) | 0.0002 |
| FPG, mmol/L | 5.4 (2.3-15.1) | 5.8 (2.3-15.1) | 5.2 (3.7-12.6) | 0.1395 |
| HbA1c, % | 5.7 (4.1-15.0) | 6.0 (4.5-9.4) | 5.6 (4.1-15.0) | 0.0016 |
| GA, % | 14.1 (4.9-31.3) | 15.1 (9.4-24.6) | 13.8 (4.9-31.3) | 0.0035 |
| ALT, U/L | 15 (3-67) | 13 (4-31) | 16 (3-67) | 0.0903 |
| AST, U/L | 16 (2-67) | 16 (6-38) | 16 (2-67) | 0.7849 |
| LDH, U/L | 206 (118-376) | 211 (132-372) | 204 (118-376) | 0.3640 |
| Scr, µmol/L | 959 (357-1813) | 920 (357-1518) | 970 (358-1813) | 0.3273 |
| BUN, mmol/L | 19.9 (8.2-37.8) | 20.1 (8.2-33.7) | 19.9 (8.4-37.8) | 0.9010 |
| UA, µmol/L | 382 (97-620) | 372 (97-543) | 384 (177-620) | 0.6291 |
| eGFR, mL/min/1.73m^2^ | 4.61 (1.90-16.16) | 4.81 (2.25-16.16) | 4.55 (1.90-14.39) | 0.3094 |
| TC, mmol/L | 4.15 (1.65-7.25) | 4.01 (1.65-7.09) | 4.20 (1.96-7.25) | 0.3012 |
| Triglyceride, mmol/L | 1.76 (0.30-9.51) | 1.65 (0.34-9.51) | 1.79 (0.30-7.83) | 0.1050 |
| LDL-C, mmol/L | 2.36 (0.44-5.26) | 2.25 (0.52-5.20) | 2.39 (0.44-5.26) | 0.3181 |
| HDL-C, mmol/L | 1.03 (0.45-5.17) | 0.98 (0.47-1.99) | 1.05 (0.45-5.17) | 0.4450 |
| Ca, mmol/L | 2.24 (1.51-2.99) | 2.22 (1.88-2.84) | 2.24 (1.51-2.99) | 0.2951 |
| P, mmol/L | 1.77 (0.56-3.29) | 1.62 (0.56-2.49) | 1.81 (0.74-3.29) | 0.0331 |
| SI, μmol/L | 13.3 (3.1-41) | 12.5 (3.1-38.5) | 13.5 (3.7-41.0) | 0.2722 |
| TSAT, % | 32 (8-93) | 31 (8-89) | 32 (8-93) | 0.6629 |
| Ferritin, ng/mL | 337.8 (12.9-2000.0) | 301.1 (17.7-972.0) | 348.4 (12.9-2000.0) | 0.6371 |
| CRP, mg/L | 7.8 (0.3-175.6) | 9.5 (0.3-63.5) | 7.3 (0.3-175.6) | 0.0244 |
| iPTH, pg/mL | 286.4 (14.3-1824) | 205.4 (14.4-1209.0) | 309.7 (14.3-1824.0) | 0.0044 |
| NT-proBNP, pg/mL | 6748.8 (92.4-35000.0) | 11181.5 (106.0-35000.0) | 5471.9 (92.4-35000.0) | ＜0.001 |
| cTnT, ng/mL | 0.080 (0.005-0.589) | 0.120 (0.017-0.589) | 0.060 (0.005-0.415) | ＜0.001 |
| D-Dimer, mg/L | 1.13 (0.15-32.60) | 1.75 (0.15-32.60) | 0.90 (0.15-11.80) | 0.0064 |
| 24-h urine volume, mL | 554 (0-2000) | 457 (0-1800) | 583 (0-2000) | 0.1018 |
| Peritoneal Kt/V | 1.51 (0.53-3.66) | 1.39 (0.58-3.34) | 1.55 (0.53-3.66) | 0.0822 |
| Renal Kt/V | 0.43 (0-2.14) | 0.28 (0-1.49) | 0.48 (0-2.14) | 0.0497 |
| Total Kt/V | 1.97 (0.78-5.00) | 1.61 (0.78-3.34) | 2.06 (1.02-5.00) | 0.0001 |
| Peritoneal Ccr, L/week/1.73m^2^ | 42.77 (16.91-132.39) | 41.86 (19.66-57.21) | 43.08 (16.91-132.39) | 0.6701 |
| Renal Ccr, L/week/1.73m^2^ | 20.65 (0-96.17) | 14.45 (0-86.55) | 22.69 (0-96.17) | 0.0619 |
| Total Ccr, L/week/1.73m^2^ | 62.86 (19.11-167.82) | 56.31 (19.66-116.03) | 65.02 (19.11-167.82) | 0.0724 |
| Right IMT, mm | 0.8 (0.4-1.3) | 0.8 (0.6-1.2) | 0.8 (0.4-1.3) | 0.2967 |
| Left IMT, mm | 0.8 (0.5-1.3) | 0.9 (0.6-1.2) | 0.8 (0.5-1.3) | 0.3669 |
| LVEF, % | 63 (34-73) | 61 (34-73) | 64 (40-72) | 0.2291 |
| LVEDD, mm | 48 (30-74) | 49 (30-60) | 48 (33-74) | 0.1921 |

Values are presented as means ± standard deviation or medians (range) for continuous variables and count (percentage) for categorical variables. DBP, diastolic blood pressure; SBP, systolic blood pressure; RBC, red blood cell; WBC, white blood cells; NEUT, neutrophil; TB, total bilirubin; DBIL, direct bilirubin; TP, total protein; FPG, fasting plasma glucose; HbA1c, glycated hemoglobin; GA, glycated albumin; Scr, serum creatinine; BUN, blood urea nitrogen; UA, uric acid; eGFR, estimated glomerular filtration rate; TC, total cholesterol; LDL-C, low-density lipoprotein cholesterol; HDL-C, high-density lipoprotein cholesterol; Ca, calcium; P, phosphorus; SI, serum iron; TSAT, transferrin saturation; CRP, C-reactive protein; iPTH, intact parathyroid hormone; NT-proBNP, N-terminal pro-brain natriuretic peptide; cTnT, cardiac troponin T; Ccr, creatinine clearance rate; IMT, carotid artery intima–media thickness; LVEF, left ventricular ejection fraction; LVEDD, left ventricular end-diastolic diameter.

**Table S7. Pairwise comparisons of the combined, clinical, and biomarker models**

| **Cohort** | **Comparison (A vs B)** | **Model A AUC (95% CI)** | **Model B AUC (95% CI)** | **ΔAUC (95% CI)** | **P value** | **Continuous NRI (95% CI)** | **IDI (95% CI)** |
| --- | --- | --- | --- | --- | --- | --- | --- |
| **Training** | Combined vs Clinical | 0.784 (0.703, 0.864) | 0.624 (0.492, 0.756) | 0.159 (0.055, 0.264) | 0.003 | 0.662 (0.307, 1.008) | 0.240 (0.163, 0.318) |
|  | Combined vs Biomarker | 0.784 (0.703, 0.864) | 0.666 (0.552, 0.781) | 0.117 (-0.013, 0.247) | 0.077 | 0.655 (0.294, 0.993) | 0.223 (0.139, 0.304) |
|  | Clinical vs Biomarker | 0.624 (0.492, 0.756) | 0.666 (0.552, 0.781) | -0.042 (-0.240, 0.155) | 0.675 | 0.089 (-0.274, 0.452) | -0.017 (-0.073, 0.034) |
| **Internal validation** | Combined vs Clinical | 0.781 (0.655, 0.907) | 0.725 (0.583, 0.868) | 0.056 (-0.018, 0.130) | 0.141 | 0.929 (0.411, 1.393) | 0.219 (0.107, 0.323) |
|  | Combined vs Biomarker | 0.781 (0.655, 0.907) | 0.612 (0.449, 0.775) | 0.170 (-0.012, 0.351) | 0.066 | 0.893 (0.375, 1.357) | 0.221 (0.104, 0.332) |
|  | Clinical vs Biomarker | 0.725 (0.583, 0.868) | 0.612 (0.449, 0.775) | 0.114 (-0.110, 0.338) | 0.320 | -0.125 (-0.571, 0.268) | 0.003 (-0.017, 0.020) |
| **External validation** | Combined vs Clinical | 0.822 (0.718, 0.927) | 0.780 (0.666, 0.894) | 0.042 (-0.042, 0.127) | 0.328 | 0.800 (0.306, 1.294) | 0.251 (0.139, 0.368) |
|  | Combined vs Biomarker | 0.822 (0.718, 0.927) | 0.770 (0.664, 0.877) | 0.052 (-0.080, 0.184) | 0.442 | 0.753 (0.235, 1.247) | 0.217 (0.122, 0.311) |
|  | Clinical vs Biomarker | 0.780 (0.666, 0.894) | 0.770 (0.664, 0.877) | 0.010 (-0.155, 0.174) | 0.908 | -0.353 (-0.800, 0.024) | -0.034 (-0.100, 0.013) |

Training, n = 168 with 37 events; internal validation, n = 72 with 16 events; external validation, n = 102 with 17 events. The outcome was CVD within 1 year. Combined denotes clinical indicators plus the two glycated peptide biomarkers; Clinical denotes clinical indicators only; Biomarker denotes the two biomarkers only. Model A is listed first and Model B second. ΔAUC = AUC of Model A − AUC of Model B. AUC and ΔAUC CIs used the DeLong method. Two-sided paired DeLong P values are shown for the three comparisons within each cohort. For Model A versus Model B, continuous NRI = P(up | event) − P(down | event) + P(down | non-event) − P(up | non-event). IDI is the change in the difference between mean predicted probabilities in events and non-events.

Abbreviations: AUC, area under the receiver operating characteristic curve; CI, confidence interval; CVD, cardiovascular disease; IDI, integrated discrimination improvement; NRI, net reclassification improvement.

**Table S8. Characteristics of the multicenter external validation cohort**

| Characteristic | Overall (n=102) | Presence of CVD (n=17) | Absence of CVD (n=85) | P value |
| --- | --- | --- | --- | --- |
| Age, y | 58 (30-80) | 63 (43-80) | 57 (30-79) | 0.1909 |
| Gender, male | 52, 51.0% | 9, 52.9% | 43, 50.6% | 1.0000 |
| Body mass index, kg/m^2^ | 24.03 (17.24-31.77) | 23.73 (19.00-29.46) | 24.09 (17.24-31.77) | 0.7122 |
| Dialysis vintage, m | 52.88 (8-172) | 46.88 (10-109) | 54.22 (8-172) | 0.7731 |
| Hypertension, % | 87, 85.3% | 16, 94.1% | 71, 83.5% | 0.4555 |
| Diabetes, % | 29, 28.4% | 10, 58.8% | 19, 22.4% | 0.0059 |
| History of CVD, % | 19, 18.6% | 6, 35.3% | 13, 15.3% | 0.0827 |
| Smoking, % | 13, 12.7% | 3, 17.6% | 10, 11.8% | 0.4506 |
| Use of antihypertensive drugs,% | 87, 85.3% | 16, 94.1% | 71, 83.5% | 0.4555 |
| Use of glucose-lowering drugs, % | 26, 25.5% | 7, 41.2% | 19, 22.4% | 0.1295 |
| Use of phosphate binders, % | 41, 40.2% | 7, 41.2% | 34, 40.0% | 1.0000 |
| SBP, mmHg | 140 (98-200) | 134 (98-180) | 141 (101-200) | 0.1649 |
| DBP, mmHg | 81 (53-127) | 75 (53-100) | 83 (63-127) | 0.0307 |
| RBC, ×10^12^/L | 3.71 (2.21-5.88) | 3.75 (2.41-4.87) | 3.70 (2.21-5.88) | 0.6546 |
| Hemoglobin, g/L | 116 (65-170) | 115 (77-154) | 116 (65-170) | 0.9129 |
| Platelet, ×10^9^/L | 203 (78-398) | 208 (83-354) | 202 (78-398) | 0.4381 |
| WBC, ×10^9^/L | 6.47 (3.60-10.81) | 6.42 (3.65-10.65) | 6.48 (3.60-10.81) | 0.8308 |
| NEUT, % | 68.4 (48.0-85.4) | 69.3 (55.6-83.1) | 68.3 (48.0-85.4) | 0.7093 |
| TB, μmol/L | 7.4 (3.0-27.1) | 7.6 (3.0-27.1) | 7.3 (3.1-23.4) | 0.6726 |
| DBIL, μmol/L | 2.78 (0.9-12.2) | 3.3 (0.9-12.2) | 2.7 (1.0-8.2) | 0.4232 |
| TP, g/L | 65 (46-81) | 63 (48-79) | 66 (46-81) | 0.2451 |
| Albumin, g/L | 37 (24-49) | 37 (28-49) | 37 (24-49) | 0.5061 |
| FPG, mmol/L | 6.4 (3.3-15.4) | 6.6 (3.4-10.4) | 6.3 (3.3-15.4) | 0.2719 |
| HbA1c, % | 6.0 (4.3-15.9) | 5.9 (4.3-7.5) | 6.1 (4.5-15.9) | 0.4469 |
| GA, % | 14.2 (5.2-21.5) | 14.9(10.2-20.9) | 14.0 (5.2-21.5) | 0.8949 |
| ALT, U/L | 16 (3-58) | 16 (3-33) | 16 (4.2-58) | 0.7311 |
| AST, U/L | 16 (6-55) | 17 (8-32) | 16 (6-55) | 0.7575 |
| LDH, U/L | 236 (99-468) | 263 (196-447) | 230 (99-468) | 0.0534 |
| Scr, µmol/L | 1006 (267-1836) | 1026 (267-1558) | 1001 (386-1836) | 0.5164 |
| BUN, mmol/L | 20.0 (1.0-36.9) | 19.1 (6.6-28.7) | 20.2 (1.0-36.9) | 0.5145 |
| UA, µmol/L | 399 (162-637) | 408 (239-498) | 396 (162-637) | 0.4307 |
| eGFR, mL/min/1.73m^2^ | 4.35 (1.87-13.53) | 4.33 (1.87-13.53) | 4.35 (2.30-9.90) | 0.3249 |
| TC, mmol/L | 4.46 (2.42-6.91) | 5.39 (2.42-6.85) | 4.25 (2.64-6.91) | 0.7689 |
| Triglyceride, mmol/L | 1.83 (0.50-6.28) | 2.00 (0.53-4.92) | 1.79 (0.50-6.28) | 0.5848 |
| LDL-C, mmol/L | 2.38 (1.01-4.40) | 2.25 (1.13-3.96) | 2.41 (1.01-4.40) | 0.4705 |
| HDL-C, mmol/L | 1.02 (0.62-2.69) | 1.06 (0.62-2.56) | 1.01 (0.63-2.69) | 0.5027 |
| Ca, mmol/L | 2.20 (1.57-3.09) | 2.21 (1.57-2.68) | 2.20 (1.68-3.09) | 0.6389 |
| P, mmol/L | 1.72 (0.98-3.15) | 1.70 (0.98-2.22) | 1.72 (0.99-3.15) | 0.8910 |
| SI, μmol/L | 16.8 (4.5-47.2) | 15.7 (5.3-30.9) | 17.0 (4.5-47.2) | 0.4741 |
| TSAT, % | 32 (12-76) | 23 (16-29) | 33 (12-76) | 0.0182 |
| Ferritin, ng/mL | 243.3 (1.7-923.7) | 308.8 (14.5-760.6) | 228.6 (1.7-923.7) | 0.9913 |
| CRP, mg/L | 7.9 (0.2-229.0) | 7.9 (0.7-46.2) | 7.9 (0.2-229.0) | 0.4610 |
| iPTH, pg/mL | 347.9 (15.6-1208.0) | 303.8 (59.6-572.9) | 357.2 (15.6-1208.0) | 0.6955 |
| NT-proBNP, pg/mL | 3051.4 (17.0-35000.0) | 6143.9 (48.0-35000.0) | 2364.1 (17.0-35000.0) | 0.2793 |
| cTnT, ng/mL | 0.026 (0.001-0.160) | 0.054 (0.012-0.160) | 0.020 (0.001-0.086) | 0.0001 |
| D-Dimer, mg/L | 0.84 (0.21-3.51) | 1.06 (0.34-3.51) | 0.77 (0.21-2.37) | 0.4258 |
| 24-h urine volume, mL | 445 (0-2300) | 313 (0-1000) | 475 (0-2300) | 0.3234 |
| Peritoneal Kt/V | 1.41 (0.60-3.20) | 1.36 (0.62-2.60) | 1.42 (0.60-3.20) | 0.5371 |
| Renal Kt/V | 0.41 (0-1.72) | 0.24 (0-1.10) | 0.45 (0-1.72) | 0.0328 |
| Total Kt/V | 1.86 (1.00-3.37) | 1.56 (1.00-2.60) | 1.93 (1.09-3.37) | 0.0048 |
| Peritoneal Ccr, L/week/1.73m^2^ | 43.56 (20.10-109.80) | 39.72 (20.10-78.30) | 44.62 (22.40-109.80) | 0.4186 |
| Renal Ccr, L/week/1.73m^2^ | 18.62 (0-62.40) | 11.85 (0-45.10) | 20.05 (0-62.40) | 0.0214 |
| Total Ccr, L/week/1.73m^2^ | 61.69 (28.30-110.64) | 51.47 (28.30-78.30) | 64.07 (31.50-110.64) | 0.0134 |
| Right IMT, mm | 0.8 (0.6-0.9) | 0.8 (0.6-0.9) | 0.7 (0.6-0.9) | 0.2905 |
| Left IMT, mm | 0.8 (0.6-1.0) | 0.8 (0.7-1.0) | 0.7 (0.6-0.9) | 0.1612 |
| LVEF, % | 64 (40-77) | 61 (50-65) | 64 (40-77) | 0.0316 |
| LVEDD, mm | 48 (41-62) | 48 (44-55) | 48 (41-62) | 0.7073 |

Values are presented as means ± standard deviation or medians (range) for continuous variables and count (percentage) for categorical variables. DBP, diastolic blood pressure; SBP, systolic blood pressure; RBC, red blood cell; WBC, white blood cells; NEUT, neutrophil; TB, total bilirubin; DBIL, direct bilirubin; TP, total protein; FPG, fasting plasma glucose; HbA1c, glycated hemoglobin; GA, glycated albumin; Scr, serum creatinine; BUN, blood urea nitrogen; UA, uric acid; eGFR, estimated glomerular filtration rate; TC, total cholesterol; LDL-C, low-density lipoprotein cholesterol; HDL-C, high-density lipoprotein cholesterol; Ca, calcium; P, phosphorus; SI, serum iron; TSAT, transferrin saturation; CRP, C-reactive protein; iPTH, intact parathyroid hormone; NT-proBNP, N-terminal pro-brain natriuretic peptide; cTnT, cardiac troponin T; Ccr, creatinine clearance rate; IMT, carotid artery intima–media thickness; LVEF, left ventricular ejection fraction; LVEDD, left ventricular end-diastolic diameter.

**Table S9. Number and reasons for participant dropout**

|  | Internal derivation cohort | External validation cohort |
| --- | --- | --- |
| Conversion to HD | 24 | 4 |
| Conversion to combined PD and HD therapy | 3 | 0 |
| Kidney transplantation | 5 | 3 |
| Death due to peritonitis | 3 | 1 |
| Death due to pneumonia | 2 | 1 |
| Death due to unknown cause | 5 | 1 |
| Lost to follow-up | 19 | 1 |
| Total dropouts | 61 | 11 |

HD, hemodialysis; PD, peritoneal dialysis.

**Table S10. Composition of cardiovascular disease events in three cohorts**

|  | **Training** | **Internal validation** | **External validation** |
| --- | --- | --- | --- |
| Acute myocardial infarction | 8 | 3 | 2 |
| Coronary artery disease | 3 | 3 | 3 |
| Cerebrovascular accident | 17 | 6 | 7 |
| Transient ischemic attack | 3 | 1 | 1 |
| Sudden cardiac death | 3 | 1 | 3 |
| Heart failure | 2 | 1 | 1 |
| Peripheral artery disease | 1 | 1 | 0 |
| Total | 37 | 16 | 17 |

**Table S11. Predictive values and Brier scores of the three models in validation cohorts**

| **Cohort** | **Model** | **PPV (95% CI)** | **NPV (95% CI)** | **Brier score (95% CI)** | **Calibration-in-the-large** | **Calibration slope** |
| --- | --- | --- | --- | --- | --- | --- |
| Internal validation | Combined | 0.429  (0.265, 0.609) | 0.909  (0.788, 0.964) | 0.186 (0.142,0.235) | -1.157 (-1.876,-0.572) | 0.896  (0.408,1.825) |
|  | Clinical | 0.400  (0.234, 0.593) | 0.872  (0.748, 0.940) | 0.244 (0.241,0.247) | -1.239 (-1.916,-0.744) | 12.883  (3.957,25.594) |
|  | Biomarker | 0.444  (0.189, 0.733) | 0.810  (0.696, 0.888) | 0.234 (0.228,0.240) | -1.154 (-1.830,-0.654) | 5.783  (-3.181,12.250) |
| External validation | Combined | 0.450  (0.258, 0.658) | 0.902  (0.819, 0.950) | 0.135 (0.107,0.166) | -1.129 (-1.797,-0.629) | 1.294  (0.784,2.157) |
|  | Clinical | 0.391  (0.222, 0.592) | 0.899  (0.813, 0.948) | 0.241 (0.239,0.244) | -1.577 (-2.185,-1.103) | 21.894  (11.739,36.582) |
|  | Biomarker | 0.429  (0.158, 0.750) | 0.853  (0.768, 0.910) | 0.223 (0.216,0.230) | -1.530 (-2.134,-1.068) | 4.052  (-0.066,13.999) |

Abbreviations: CI, confidence interval; CVD, cardiovascular disease; PPV, positive predictive value; NPV, negative predictive value.

Table S12. Covariate-adjusted associations **per interquartile-range increase** of glycated peptide

with 1-year CVD in the derivation cohort

| **Adjustment model** | **GEAFTLK(g)ATVLNYLPK** | | **K(g)QHLFVK** | |
| --- | --- | --- | --- | --- |
|  | **OR (95% CI)** | **P value** | **OR (95% CI)** | **P value** |
| M0: Unadjusted | 2.181 (1.273–3.831) | 0.004 | 2.279 (1.327–4.018) | 0.003 |
| M1: adjusted for age, sex, diabetes, previous CVD | 2.040 (1.142–3.735) | 0.016 | 1.977 (1.116–3.581) | 0.019 |
| M2: M1 + renal Ccr | 2.801 (1.275–6.622) | 0.010 | 2.044 (1.002–4.298) | 0.049 |
| M3: M1 + total Kt/V | 2.226 (1.059–4.900) | 0.034 | 1.956 (0.954–4.131) | 0.067 |
| M4: age, sex, previous CVD + glucose-lowering drugs, antihypertensive drugs and phosphate binders | 1.961 (1.076–3.662) | 0.028 | 1.690 (0.933–3.105) | 0.084 |
| M5: M1 + phosphate, GA and albumin | 2.021 (1.089–3.872) | 0.026 | 2.075 (1.162–3.797) | 0.013 |
| M6: M1 + renal Ccr + peritoneal Kt/V | 2.657 (1.199–6.333) | 0.016 | 2.024 (0.983–4.304) | 0.056 |

**Figure S1. ROC curves of the four candidate glycated peptide biomarkers individually and in combination**

The colored curves show the discrimination of individual peptides and their combination between participants with and without CVD. The red curve labeled “All” represents the combination of all four peptides. Areas under the ROC curve are shown in parentheses in the legend. The black dashed line indicates chance-level discrimination.

**Figure S2. Calibration of the combined, clinical-only, and biomarker-only models in the internal and external validation cohorts**

Calibration plots for predicting 1-year cardiovascular disease (CVD) risk are shown for the internal validation cohort (A, C, and E) and the external validation cohort (B, D, and F). Figure A and B represent the combined model, C and D represent the clinical-only model, and E and F represent the biomarker-only model. Solid blue lines indicate flexible calibration curves, with shaded areas representing pointwise 95% bootstrap confidence intervals. Gray dashed diagonal lines indicate ideal agreement between predicted and observed risks. Open circles represent observed CVD event rates within quartiles of predicted risk, with vertical error bars indicating 95% Wilson confidence intervals. The stacked histograms beneath each plot show the distribution of predicted probabilities, with red and gray segments representing patients with and without CVD events, respectively.

CI, confidence interval.

**Figure S3. Decision curve analysis of three models in the validation cohorts**

Net benefit across threshold probabilities is shown for the internal testing set (left) and external validation cohort (right). Red, green, and blue curves represent the biomarker-only, clinical-only, and combined models, respectively. The gray solid line and black dashed line represent the treat-all and treat-none strategies, respectively.
